# Septins buffer actomyosin forces to protect the nucleus from genotoxic mechanical stress

**DOI:** 10.1083/jcb.202602074

**Published:** 2026-10-22

**Authors:** Franklin Mayca Pozo, Sarah M. Butts, Andrew W. Schaefer, Cristina Montagna, Elias T. Spiliotis

**Affiliations:** 1 Department of Cell Biology, University of Virginia School of Medicine, Charlottesville, VA, USA; 2 Department of Radiology and Oncology, Rutgers Cancer Institute of New Jersey, New Brunswick, NJ, USA

## Abstract

Invasively migrating cells thread their nucleus through confined interstitial spaces. How cells protect the nucleus from intracellular forces is poorly understood. Here, we show that the septin cytoskeleton buffers the actomyosin forces that have been shown to power nuclear movement. Septin filaments comprising SEPT9, a septin amplified in breast cancer, align with perinuclear actomyosin cables, which exhibit higher tensile stress during 3D confined migration through narrower pores. SEPT9 depletion amplifies actin stress during confined migration and after myosin II hyperactivation in nonmigrating cells causing actin and nuclear envelope ruptures. Following confined migration, DNA breaks, nuclear blebs, micronuclei, and cell death increase in SEPT9-depleted cells, phenotypes rescued by the oncogenic SEPT9 isoform 1. Clinicogenomic data reveal that *SEPT9* amplification associates with lower genomic alteration in aggressive breast tumors. We propose that septins are a mechanoprotective element of the cytoskeleton, and SEPT9 amplification enhances tumor cell survival by preventing nuclear damage.

## Teaser

Mayca Pozo et al. show that septin filaments are a mechanoprotective cytoskeletal element, buffering actomyosin forces to cushion the nucleus during confined migration. Cells depleted of SEPT9_i1 suffer nuclear damage, DNA breaks, and death. This mechanoprotective role may provide a survival advantage to aggressive breast cancers with SEPT9 amplification by averting genomic catastrophe during invasive migration.

## Introduction

Cell migration is a fundamental biological process underlying organismal development, tissue homeostasis and regeneration, immunity, and cancer metastasis (Friedl and Gilmour, 2009; Masopust and Schenkel, 2013). In the 3D environment of tissues and interstitial spaces, cell migration is physically constrained by the dimensions of the surrounding microenvironment (Yamada and Sixt, 2019). The nucleus, the largest and most rigid organelle in the cell, poses a major physical impediment that restricts cell mobility (Calero-Cuenca et al., 2018; Lele et al., 2018). Cells overcome this limitation by modifying the physical properties of the nucleus and/or the extracellular matrix (ECM) (Cambi and Chavrier, 2021; Cho et al., 2017; Friedl et al., 2011; Miroshnikova and Wickström, 2022) and by employing different strategies and modes of migration (Alonso-Matilla et al., 2025; Gardel et al., 2010; Petrie and Yamada, 2016).

The nucleus is a mechanosensitive organelle, sensing not only external spatial constraints (Lomakin et al., 2020; Renkawitz et al., 2019; Venturini et al., 2020), but also forces transmitted from the ECM through the cytoskeleton (Cho et al., 2017; Kim et al., 2018; Lele et al., 2018; Miroshnikova and Wickström, 2022). Nuclear mechanosensitivity is a central determinant of cell behavior and identity, as gene expression and nucleocytoplasmic transport are directly impacted by forces acting on chromatin and nuclear pores (Bellas and Chen, 2014; Buchwalter et al., 2019; Dupont and Wickström, 2022). While advantageous for cellular homeostasis, nuclear mechanosensitivity poses a risk to nuclear and genome integrity. During confined migration, the nucleus is subjected to multiple forces (e.g., contractile, frictional, hydrostatic), which collectively impose mechanical stresses capable of rupturing the nuclear envelope and damaging genomic DNA (Kalukula et al., 2022; de Freitas Nader et al., 2021). These injuries alter nucleocytoplasmic transport and risk DNA leakage into the cytoplasm, potentially leading to further genomic alterations, cell death, or the pathogenesis of diseases such as autoimmunity and cancer (Lim et al., 2016; Mammel and Hatch, 2022; Tubbs and Nussenzweig, 2017).

How cells mitigate mechanical stress to avoid nuclear damage during confined migration is not well understood. In the nucleoplasm, lamin intermediate filaments provide structural support and determine nuclear stiffness, dictating how nuclei respond to mechanical stress and deformation (Davidson et al., 2014; Friedl et al., 2011; Sobo et al., 2024). Softer nuclei with lower levels of lamin A/C are more amenable to confined migration but are also susceptible to nuclear damage, which may be compensated by as-yet-unidentified factors (Bell et al., 2022; Harada et al., 2014; Singh et al., 2013). In the cytoplasm, vimentin filaments form a cage-like structure around the nucleus, protecting it from rupture by reinforcing perinuclear stiffness while absorbing contractile forces (Patteson et al., 2019). Recent work suggests that vimentin functions in conjunction with perinuclear microtubules to buffer compressive forces (Alisafaei et al., 2024; Ju et al., 2024). However, vimentin also promotes the tensile forces of cell migration, which are powered by actomyosin filaments anchored to focal adhesions (Guo et al., 2025; Swoger et al., 2025). Vimentin’s dual role in absorbing and transmitting forces, along with its context-dependent effects as either an inhibitor or enhancer of cell migration, points to additional mechanisms of force regulation that remain to be elucidated.

Septins comprise a distinct network of nonpolar cytoskeletal polymers that assemble from multimeric GTP-binding proteins (Cavini et al., 2021; Mostowy and Cossart, 2012). Septins associate with subsets of actin filaments and microtubules, as well as specific membrane subdomains, regulating the spatial organization of cellular proteins and processes (Benoit et al., 2023; Spiliotis and Nakos, 2021; Woods and Gladfelter, 2021). Septin 9 (SEPT9) is ubiquitously expressed and mediates the interaction of septin oligomers and polymers with actin and microtubules (Bai et al., 2013; Dolat et al., 2014; Iv et al., 2021; Kuzmić et al., 2022; Smith et al., 2015). SEPT9 isoform 1 (SEPT9_i1) is the longest SEPT9 isoform and possesses actin-binding and crosslinking activity (Dolat et al., 2014; Martins et al., 2023; Smith et al., 2015). SEPT9_i1 has been functionally implicated in angiogenesis (Amir et al., 2010), cell migration (Connolly et al., 2011; Dolat et al., 2014; Farrugia et al., 2020; Hecht et al., 2024; Zeng et al., 2019), and ECM degradation by invadosomes (Marcus et al., 2019; Okletey et al., 2023). Notably, overexpression and depletion of SEPT9_i1 enhances and reduces, respectively, tumor growth and metastasis in vivo (Xu et al., 2018; Zeng et al., 2019; Zhang et al., 2020).

The mechanical properties of septins in cell migration and broader cell biology remain largely unexplored. Recent work indicated that SEPT9_i1 influences nuclear deformability, impacting the formation of invadopodia (Okletey et al., 2023), which occurs partly in response to the inability of the nucleus to overcome physical confinement (Infante et al., 2018; Wolf et al., 2013), and may involve a mechanical crosstalk between the nuclear and plasma membrane ends of invadopodia (Revach et al., 2015). However, whether septins function in nuclear mechanosensation and/or mechanotransduction during confined cell migration remains unknown. Here, we report an unexpected role for septins in relieving actomyosin-generated forces, which have been shown to promote nuclear movement during cell migration through pores smaller than the nucleus (Beach et al., 2011; Beadle et al., 2008; Harada et al., 2014; Thomas et al., 2015; Thomas et al., 2016). As myosin contractility can compromise nuclear and genomic integrity (Hatch and Hetzer, 2016; Takaki et al., 2017; Xia et al., 2019), SEPT9_i1 protects both actin filaments and the nucleus from actomyosin-generated forces. In 3D transmigration and 2D myosin hyperactivation assays, SEPT9_i1 depletion enhances actin and nuclear damage, and reduces cell viability during cell migration through narrow pores. Clinicogenomic analyses reveal that *SEPT9* copy-number amplification associates with distinct thresholds of genomic alteration in aggressive breast cancer tumors. We propose that *SEPT9* amplification favors the survival of cancer cells with elevated genomic instability by buffering mechanical stress during confined migration, thereby preventing genomic catastrophe and facilitating metastatic progression.

## Results

### Septin filaments align with contractile actomyosin cables in cellular regions of confined nuclear movement

**Figure 1.**
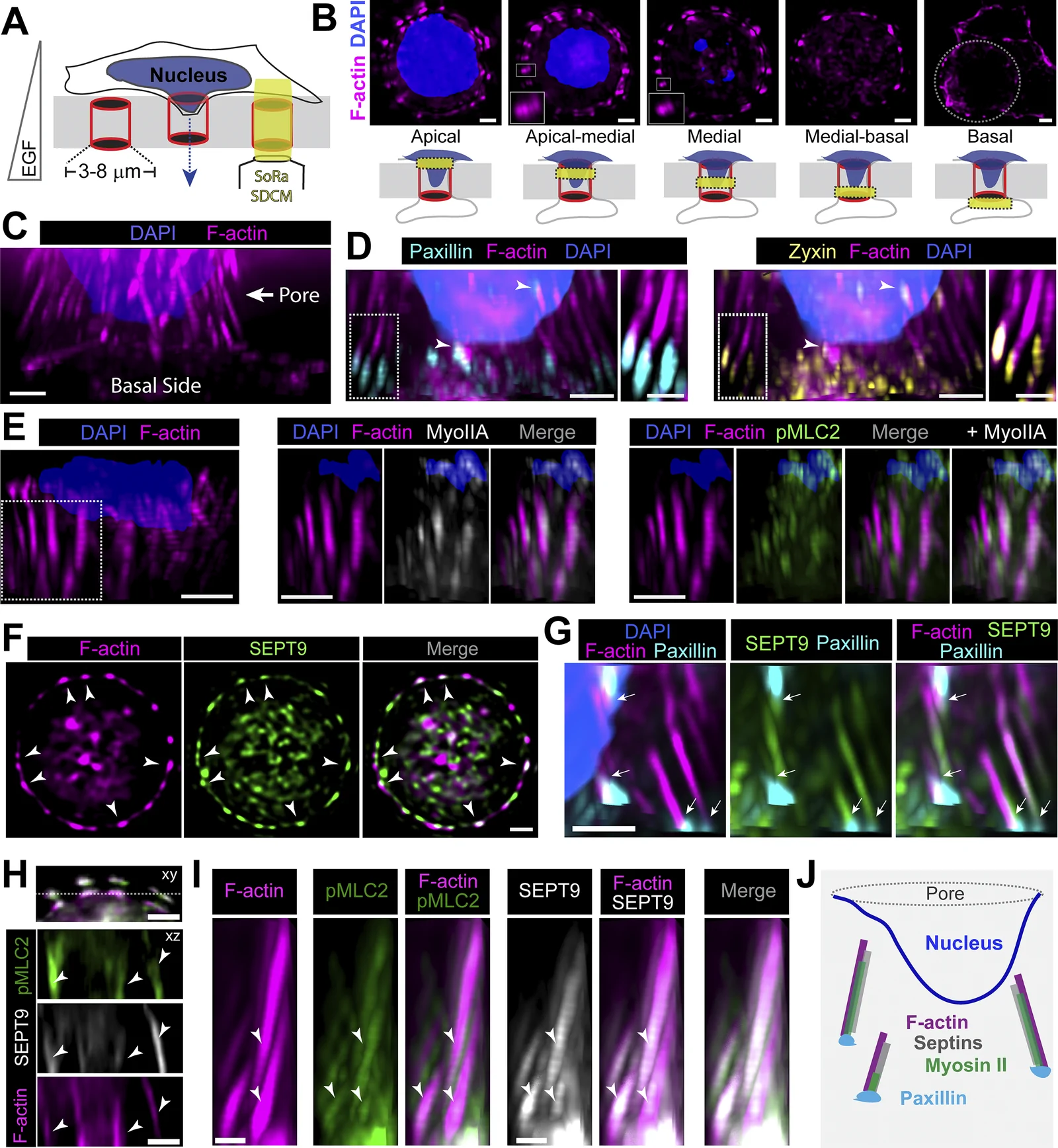
**Septins align with perinuclear contractile actomyosin cables during confined cell migration. (A)** Schematic of the experimental configuration for imaging confined cell migration in 3D. Transwell polycarbonate membranes with pores of variable diameter were coated with ECM, and U2OS cells migrated through the pores by chemotaxis along an EGF gradient. Cellular regions within the pore were imaged by super-resolution SoRa SDCM. **(B)** Maximum-intensity projections of cross-sectional images from ∼1-μm-thick regions at apical, apical–medial, medial, medial–basal, and basal depths of the pore, indicated by the yellow band in the schematic below each fluorescence image. Insets show magnified views of phalloidin-stained F-actin clusters (groups of vertical filaments, magenta) surrounding the confined nuclear mass (DAPI, blue) with semi-regular spacing along the pore circumference. The basal cross section shows cortical actin at the cell membrane extending from the pore (dotted circle) to the basal surface of the porous membrane. Scale bars, 1 μm. **(C)** Side view of a 3D volume showing F-actin (phalloidin, magenta) relative to the confined nucleus (DAPI, blue) within the pore and the plasma membrane extending onto the basal surface. Scale bars, 1 μm. **(D)** Sagittal view of 3D-rendered images of the confined nucleus (DAPI, blue), F-actin (phalloidin, magenta), paxillin (cyan), and zyxin (yellow). Scale bars, 1 μm (0.5 μm in magnified inset). **(E)** Sagittal views of 3D-rendered images showing myoIIA (grayscale) and pMLC2 (green) localization to F-actin cables (phalloidin, magenta) in the perinuclear region outlined in the leftmost panel (dashed rectangle). Scale bars, 1 μm. **(F)** Medial–basal optical section showing F-actin (phalloidin, magenta) and SEPT9 (green) colocalization (arrowheads) along the pore circumference. Arrows point to F-actin that colocalizes with SEPT9. Scale bar, 1 μm. **(G)** Sagittal view of perinuclear actin cables (phalloidin, magenta) colocalizing with SEPT9 (green) and contacting paxillin (cyan; arrows). Scale bar, 1 μm. **(H)** SoRa SDCM images show single optical xy (top panel) and xz sections across the plane outlined with a dashed line. In the top panel, F-actin (phalloidin, magenta), SEPT9 (grayscale), and pMLC2 (green) are merged. Arrowheads point to actin segments with pMLC2 and SEPT9. Scale bars, 1 μm. **(I)** Side view of a subcellular region stained for F-actin (phalloidin, magenta), pMLC2 (green), and SEPT9 (grayscale) after 3D rendering. Arrowheads point to actin segments with pMLC2 and SEPT9. Scale bars, 0.5 μm. **(J)** Schematic illustrates the organization of perinuclear septoactomyosin cables in regions of confined nuclear movement. All U2OS cell images were acquired with super-resolution SoRa SDCM 12 h after plating on 8-μm-wide pores. In 3D views, occasional banding of F-actin fluorescence was due to sub-Nyquist sampling, which was necessary to offset photobleaching during acquisition of large image z-stacks. SDCM, spinning-disk confocal microscopy.

We sought to investigate how septins function during confined nuclear movement. We used a 3D assay of cell migration through micron-scale (3–8 μm wide) pores of track-etched polymeric membranes, which were coated with ECM (Matrigel/laminin) (Fig. 1 A). Bathed in media from both apical and basal sides, the 10- to 15-μm-deep pores serve as interstitial-like conduits of chemotactic migration along a concentration gradient of epidermal growth factor (EGF). We combined this biomimetic platform with 3D super-resolution SoRa spinning-disk confocal microscopy, which provided an unprecedented view and resolution of the cellular regions within the micron-scale environment of ECM-containing pores.

**Figure S1.**
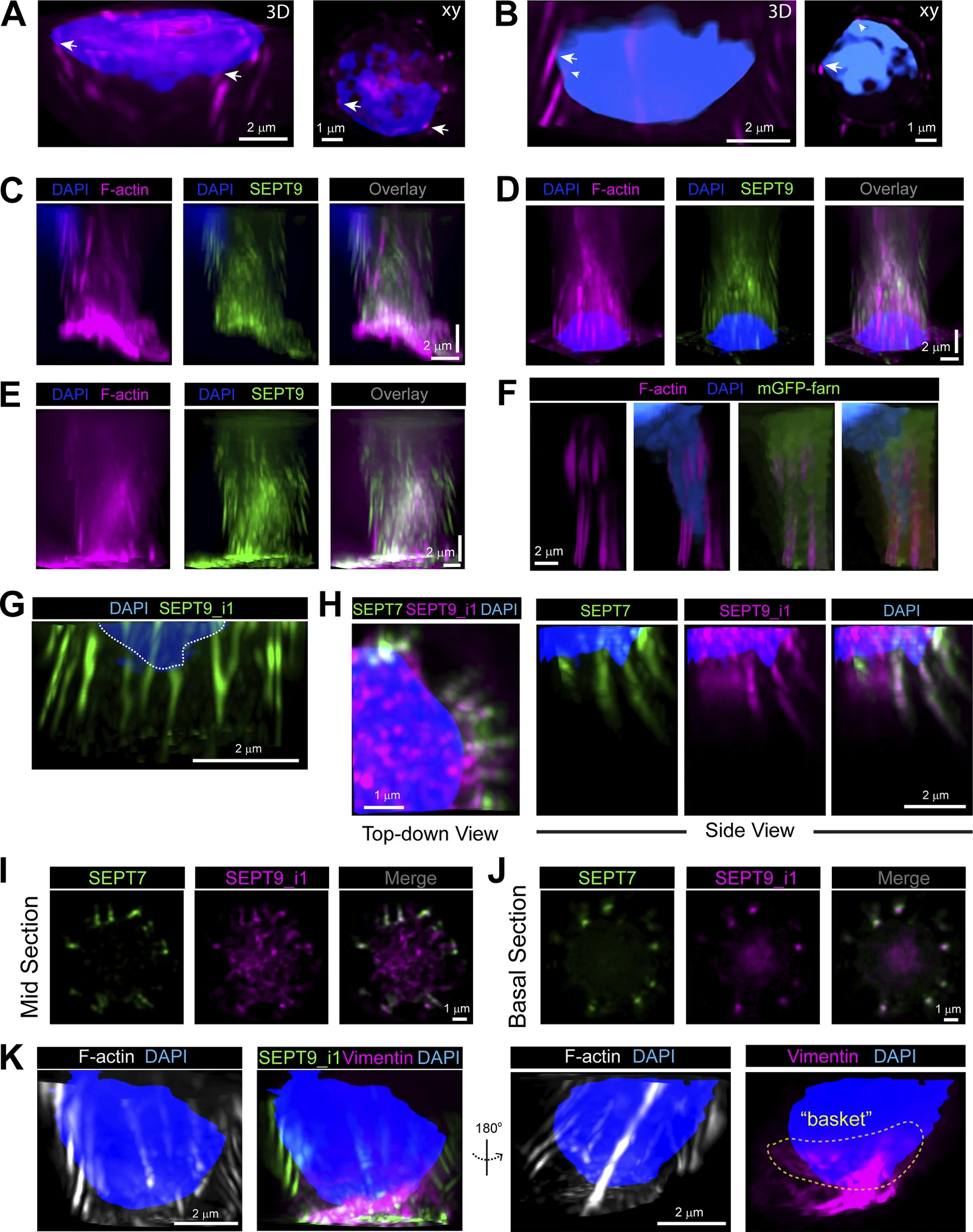
**Perinuclear septin cables consist of SEPT9_i1 and SEPT7, and are spatially distinct from the nuclear vimentin basket. (A and B)** Images show 3D side views (left) and single 2D (xy) optical sections (right) of F-actin (phalloidin, magenta) and the nucleus (blue, DAPI) of a U2OS cell in early (A) and later (B) stages of movement through an 8-μm-wide pore. Arrows point to actin cables which appear to contact the nucleus. Scale bars, 2 μm (3D image) and 1 μm (xy image). **(C and D)** Sagittal views of 3D-rendered images of U2OS cell regions in 8-μm-wide pores with nuclei in early (C) and late (D) stages of transmigration. Images show F-actin (phalloidin, magenta), SEPT9 (green), and the nucleus (DAPI, blue). Scale bars, 2 μm. **(E)** Sagittal views of 3D reconstructed images of an intrapore U2OS cell region that lacks the nucleus stained for F-actin (phalloidin, magenta) and SEPT9 (green). Scale bars, 2 μm. **(F)** Sagittal 3D view of actin cables (phalloidin, magenta), plasma membrane (mGFP-farn, green), and the nucleus (DAPI) of a U2OS cell region at the edge of the inner rim of an 8-μm-wide pore (see also Video 2). Scale bar, 2 μm. **(G)** Sagittal view of SEPT9_i1 (green) and the nucleus (DAPI, blue) in a 3D volume region of a U2OS cell confined in an 8-μm-wide pore. Scale bar, 2 μm. **(H)** Top-down and sagittal side views of a confined U2OS subcellular region after staining for SEPT7 (green), SEPT9_i1 (magenta), and the nucleus (DAPI, blue). Images were 3D-reconstructed, and volume-rendered. Scale bars, 1 μm (top-down view) and 2 μm (side view). **(I and J)** Single optical sections of a U2OS cell region taken from medial (mid; I) and basal (J) depths after staining for SEPT7 (green) and SEPT9_i1 (magenta). Scale bar, 1 μm. **(K)** Sagittal side views of the nucleus (DAPI, blue), F-actin (phalloidin, grayscale), SEPT9_i1 (green), and vimentin (magenta) in a 3D volume region of a U2OS cell confined in an 8-μm-wide pore. Two views are shown after 180° rotation around the vertical axis. The perinuclear vimentin basket is outlined with a yellow dashed line. Scale bars, 2 μm. All U2OS cell images were acquired with super-resolution SoRa SDCM 12 h after plating on 8-μm-wide pores. SDCM, spinning-disk confocal microscopy.

Imaging the actin cytoskeleton of the human osteosarcoma U2OS cells revealed an array of actin cables oriented vertically along the apicobasal axis of migration, surrounding the portion of the nucleus that has entered the pore (Fig. 1 B). These cables ran parallel to the pore-occupying nuclear mass, resembling the vertical bars of a birdcage (Fig. 1 and Video 1). Transverse optical sections taken from the apex to the base of the pore, combined with 3D reconstructions, showed that individual actin cables initiated and terminated at different pore depths, with several cables overlapping vertically one above the other in stacked groups of two, three, or more (see insets in Fig. 1, B and D; and Video 1). Within these stacks, the inner most filaments occasionally made lateral contact with the nucleus (Fig. S1, A and B, arrows). In 8-μm-wide pores, actin cables displayed a circular arrangement with semi-regular spacing (Fig. 1 B), but this symmetrical organization was not as consistent in narrower pores.

**Video 1.** **Perinuclear actin cables surround the pore-occupying nucleus and contact paxillin during 3D confined migration.** Super-resolution SoRa spinning-disk confocal microscopy of a U2OS cell region within an 8-μm-wide pore, showing F-actin (phalloidin, magenta), paxillin (green), and nucleus (DAPI, blue) after 3D volume rendering. The image rotates 180° degrees around the vertical axis of the pore. At the top of the rotating view, background fluorescence from actin filaments and the nucleus is visible from the cell region attached to the apical side of the porous Transwell membrane. The beaded appearance of some actin cables is due to sub-Nyquist sampling, which was necessary to offset photobleaching during acquisition of a large image z-stack. Image width, ∼10 μm.

**Figure S2.**
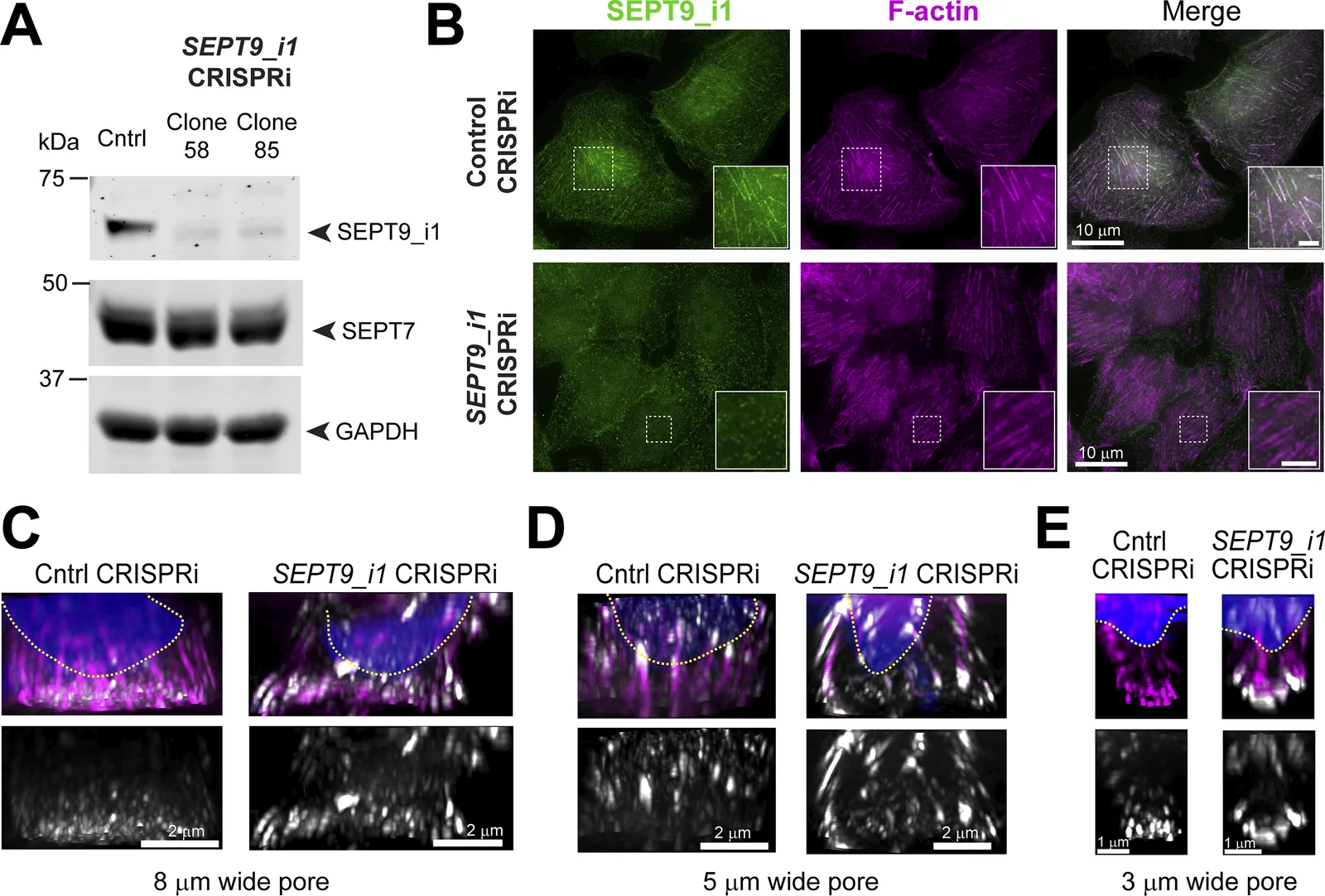
**Septin expression in genome-edited U2OS cells, and zyxin increase in confined SEPT9_i1-depleted U2OS cells. (A)** Western blots of lysates of parental control U2OS cells and cell clones 58 and 85 after selection for stable expression of SEPT9_i1-targeting sgRNAs. Cell lysates were blotted with antibodies against the SEPT9_i1 isoform, SEPT7 and GAPDH. **(B)** Wide-field microscopy images of U2OS cells that stably express control and SEPT9_i1 sgRNAs after staining for F-actin (phalloidin, magenta) and the SEPT9_i1 isoform (green). Insets show outlined regions in higher magnification. Scale bars, 10 μm (2 μm, insets). **(C–E)** Sagittal side views of 3D rendered images of the pore-occupying regions of control and SEPT9_i1-depleted U2OS cells in 8-μm-wide (C), 5-μm-wide (D), and 3-μm-wide (E) pores. Cells were stained with phalloidin (F-actin, magenta) and antibody against zyxin (grayscale). Nuclear areas are outlined with a dashed yellow line. Scale bars, 2 μm (C and D) and 1 μm (E). Source data are available for this figure: SourceData FS2.

Juxtanuclear actin cables were also prominent at sites of nuclear entry and exit (Fig. S1, C and D), but less abundant in intrapore cell regions that lacked the nucleus (Fig. S1 E). Perinuclear areas contained 2.2 ± 0.3-fold more actin cables than nuclear-free areas—66.5 ± 2.7% of total intrapore actin filaments (*n* = 10 cells). Labeling the plasma membrane with farnesylated mGFP (mGFP-farn) showed that actin cables were also closely apposed to the membrane lining the inner rim of the pore (Video 2 and Fig. S2 F). Overall, most cables were positioned near the pore’s inner rim and made lateral contacts with nucleus when it lay close to the rim. When the nucleus was more centrally positioned within the pore, contacts with a subset of inner actin cables were observed sporadically.

**Video 2.** **Perinuclear actin cables at the pore rim are closely apposed to the plasma membrane.** Super-resolution SoRa spinning-disk confocal microscopy of a U2OS cell region within an 8-μm-wide pore, showing the plasma membrane (mGFP-farn, green), F-actin (phalloidin, magenta), and nucleus (DAPI, blue) after 3D volume rendering. The image rotates 180° around the vertical axis of the pore with gradual disappearance of the nucleus and actin cables, and sequential restoring of the actin cables with the nucleus in the absence of the cortical membrane, which is reintroduced at the end. A scale grid shows distances in the x, y, and z axes in increments of ∼2 μm.

To determine whether the perinuclear actin cables transduce forces, we examined the localization of force-sensing proteins paxillin and zyxin, which accumulate at focal adhesions and sites of actin filament strain (Smith et al., 2013; Sun et al., 2020), myosin II heavy chain (myosin IIA [myoIIA]), and the phosphorylated (pT18/pS19) myosin regulatory light chain (pMLC2), as a marker for myosin II contractility (Adelstein and Conti, 1975). Actin cables were anchored to focal concentrations of paxillin and zyxin, which localized predominantly on the basal side of the pore distal to the edge of the nucleus (Fig. 1 D, insets; Video 1). Occasionally, actin cables contacted paxillin and zyxin foci in closer proximity to the nucleus at apical and medial regions of the pore (Fig. 1 D, arrowheads). Consistent with active force transduction, actin cables were closely juxtaposed to myoIIA, which appeared as thick cable-like filaments similar to actin, as well as pMLC2, which localized to patches and segments along the actomyosin cables (Fig. 1 E). Together, these findings suggest that the nucleus is surrounded by contractile actomyosin cables that generate forces that may promote nuclear movement in the apicobasal direction of migration.

We next examined the localization of septin filaments and vimentin intermediate filaments, which associate with and function synergistically with actomyosin during cell migration (Guo et al., 2025; Spiliotis and Nakos, 2021; Swoger et al., 2025). We immunostained for SEPT9 and SEPT7, which are ubiquitous and requisite subunits of the octameric septin protomer (Cavini et al., 2021). Using SEPT9 pan-isoform and SEPT9_i1 isoform-specific antibodies, we found that septin filaments exhibited similar localization and distribution to perinuclear actomyosin cables (Figs. 1 F, S1, C, D, and G; and Video 3). SEPT9_i1 filaments were closely juxtaposed to actin cables, overlapping laterally along their vertical length (Fig. 1 G and Video 3). SEPT9 was present on actin cables that connected to paxillin foci (Fig. 1 G, arrows) and contained pMLC2 (Fig. 1, H and I), indicating that septins are an integral component of the actomyosin mechanotransduction machinery (Fig. 1 J). The percentage of actin cables per cell in contact with paxillin was 78 ± 5%, and the percentage of SEPT9-coated actin cables terminating to paxillin was 88 ± 4% (*n* = 23 cells).

**Video 3.** **SEPT9_i1-containing filaments align vertically with actin cables that surround the pore-occupying nuclear mass.** Super-resolution SoRa spinning-disk confocal microscopy of a U2OS cell region within an 8-μm-wide pore, showing F-actin (phalloidin, magenta), SEPT9_i1 (green), and nucleus (DAPI, blue) after 3D volume rendering. Image rotates 180° around the vertical axis of the pore. The banded appearance of some actin cables is due to the sub-Nyquist sampling during the acquisition of a large image z-stack, which was applied to minimize photobleaching. Image width, ∼8 μm.

Consistent with septin filaments composed of the canonical SEPT2/6/7/9 protomer, SEPT7 exhibited similar localization to SEPT9 colocalizing with the oncogenic SEPT9_i1 isoform (Fig. S1, H–J). In contrast, vimentin differed markedly in its localization from the septoactomyosin cables. Vimentin intermediate filaments accumulated predominantly on the basal side of the nucleus, forming a basket-like organization around the ventral nuclear edge and a dense meshwork along the ventral plasma membrane emerging from the basal side of the pore (Fig. S1 K). Perinuclear vimentin filaments with an organization resembling the vertical septoactomyosin cables were scarce. These results reveal that during confined migration, septin filaments align with contractile actomyosin cables in a birdcage-like perinuclear arrangement that is distinct from the vimentin cytoskeleton.

### Septins buffer actomyosin forces, protecting actin cables from increasing mechanical strain in narrower pores

Given that the actomyosin cables that surround the pore-occupying nucleus exhibit active mechanotransduction, we investigated how they respond to mechanical strain and how septins function in this process. We reasoned that if actomyosin cables actively drive nuclear threading, they would experience increasing mechanical tension in narrower pores, as higher forces might be needed to move the nucleus forward—due to greater physical constraint and possibly fewer actomyosin cables owing to reduced cytoplasmic volume. To test this prediction, we decreased stepwise the diameter size of Matrigel-filled pores and imaged zyxin in U2OS cell regions that occupied 8-, 5-, and 3-μm-wide pores. We also generated stable clones of SEPT9_i1-depleted U2OS cells using CRISPR/Cas9 to assess whether septins and more specifically SEPT9_i1 impact the mechanical strain of confined migration (Fig. S2, A and B).

**Figure 2.**
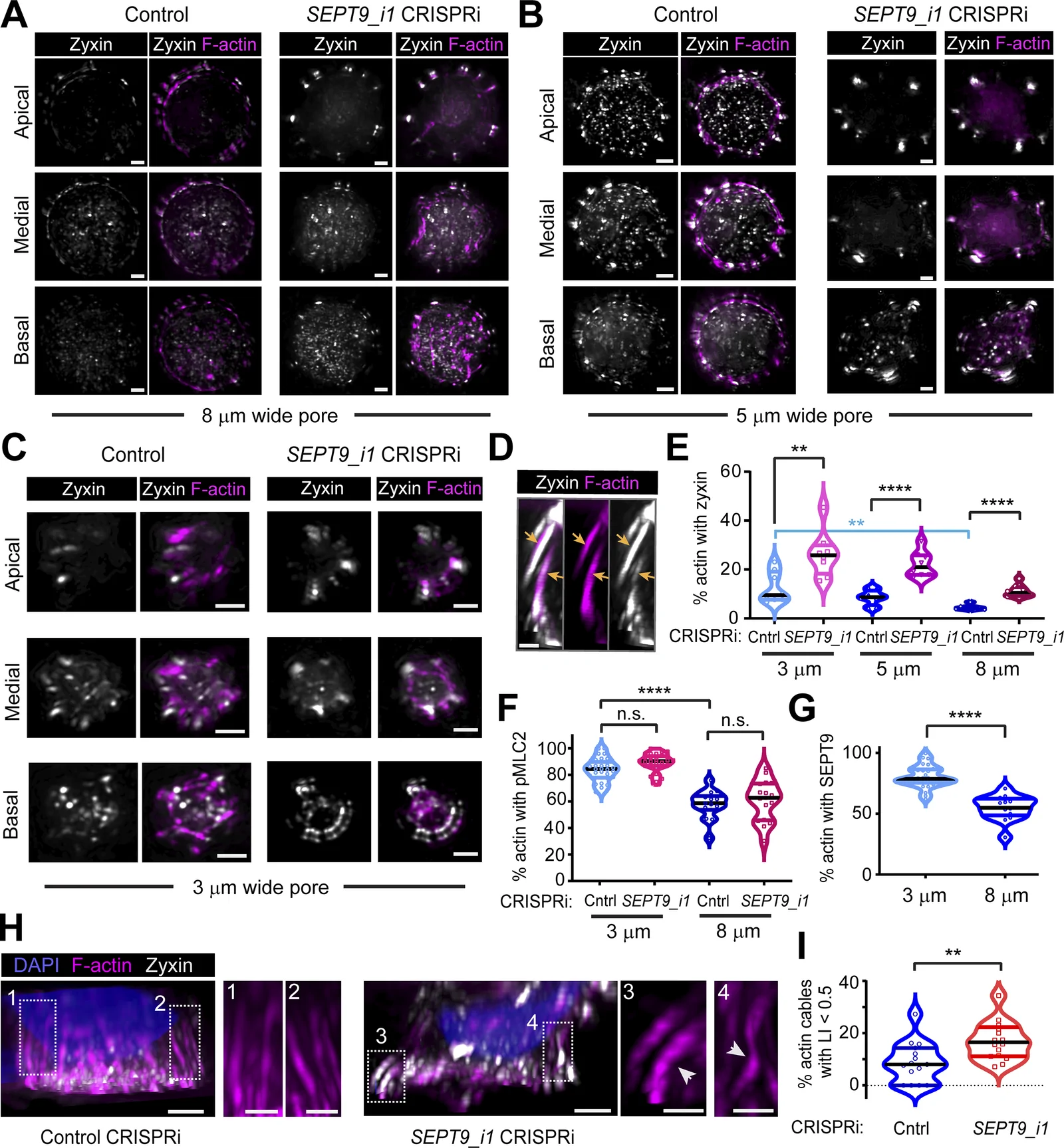
**Septins reduce mechanical stress on the actomyosin cytoskeleton during migration through increasingly narrow pores. (A–C)** Maximum-intensity projections of cross-sectional images from ∼1-μm-thick regions at apical, medial, and basal depths of 8-μm-wide (A), 5-μm-wide (B), and 3-μm-wide (C) pores. The pore-occupying regions of genome-edited U2OS cells (parental control and SEPT9_i1 CRISPRi) are shown after staining for F-actin (phalloidin, magenta) and zyxin (grayscale). Scale bars, 1 μm. **(D)** Sagittal view of a subcellular 3D volume region of a SEPT9_i1-depleted U2OS cell confined in a 5-μm-wide pore. Arrows point to zyxin (grayscale) localization along the length of actomyosin cables (phalloidin, magenta). Scale bar, 0.5 μm. **(E)** Violin plots show the percentage of zyxin-decorated actin cables in genome-edited U2OS cell (parental control and SEPT9_i1 CRISPRi) regions confined in 3 μm (*n* = 96–102 cables; eight cells), 5 μm (*n* = 185–190 cables; eight cells), and 8 μm (*n* = 381–390 cables; eight cells) pores. Statistical analysis between the indicated pair groups was done with Welch’s *t* test. **, P < 0.01; ****, P < 0.0001. **(F)** Violin plots show the percentage of actin cables coated with pMLC2 as the mean value per cell. Quantifications were done in U2OS cell regions in 3-μm pores (*n* = 31 parental control cells; *n* = 28 SEPT9_i1 CRISPRi cells) and 8-μm pores (*n* = 15 parental control cells; *n* = 17 SEPT9_i1 CRISPRi cells). Statistical comparisons between the indicated groups were done with a nonparametric Kruskal–Wallis one-way ANOVA with Dunn’s multiple comparisons test. ****, P < 0.0001; n.s., not significant (P > 0.05). **(G)** Violin plot shows the percentage of actin cables coated with SEPT9 as the mean value per U2OS cell region in 3-μm-wide (*n* = 31 cells) and 8-μm-wide (*n* = 15 cells) pores. Statistical analysis was done with Welch’s *t* test. ****, P < 0.0001. **(H)** Sagittal view of 3D volume regions of control and SEPT9_i1-depleted U2OS cells confined in 8-μm-wide pores after staining for actin (phalloidin, magenta) and nuclei (DAPI, blue). Insets show in higher magnification the actin cables outlined in dashed rectangles. Arrowheads point to bent actin filaments with high curvatures. Scale bars, 1 and 0.5 μm (inset). **(I)** Violin plots show the percentage of actin filaments (>2 μm long) per U2OS cell region within an 8-μm-wide pore with a LI below 0.5. Quantifications were done for parental control and SEPT9_i1 CRISPRi cells (*n* = 15 cells), and analyzed with Welch’s *t* test. **, P < 0.01. All violin plots show the median (black line), and first and third quartiles (colored lines). All images were acquired with super-resolution SoRa SDCM in pores with diameters of 8-μm-, 5-μm-, and 3-μm-wide pores 12, 24, and 36 h, respectively, after seeding. SDCM, spinning-disk confocal microscopy.

**Figure S3.**
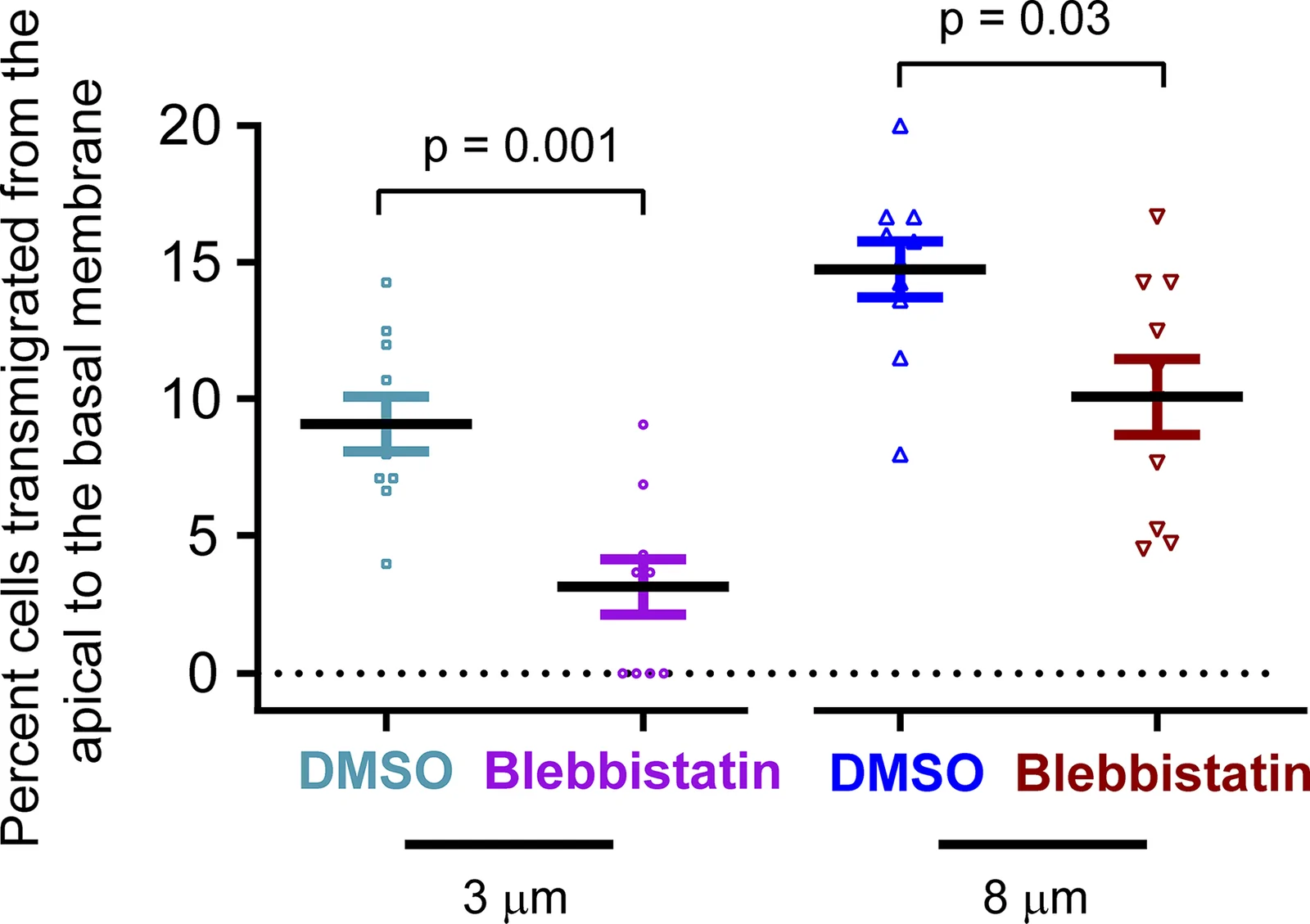
**Inhibition of myosin II activity with blebbistatin impedes pore-restricted U2OS migration.** The graph shows the percentage of U2OS cells that transmigrated fully from the apical to the basal membrane of Transwell membranes with 8-μm-wide and 3-μm-wide pores. Completion of transmigration was assessed by lack of nuclear (DAPI) and actin filament (phalloidin) stain inside the delimiting pore in 3D image stacks obtained by spinning-disk confocal microscopy. 10 fields of view (*n* = 10) were analyzed for DMSO (256 cells in total) and blebbistatin (5 μM; 251 cells in total) treatments after 12 h of transmigration through 8-μm-wide pores. 10 fields of view (*n* = 10) were also analyzed for DMSO (220 cells in total) and blebbistatin (5 μM; 202 cells) treatments after 24 h of transmigration through 3-μm-wide pores. Statistical analysis was performed with a Brown–Forsythe and Welch ANOVA test (P < 0.0001) with a Dunnett T3 multiple comparisons test. P = 0.001 (3 μm-wide pores); P = 0.03 (8 μm-wide pores).

In optical sections from the apical, medial, and basal regions of the pore, zyxin levels appeared to increase with decreasing pore diameters (Figs. 2, A–C and S2, C–E). We quantitatively assessed whether actin cables were subjected to higher mechanical strain by measuring the percentage of zyxin-decorated actin cables per cell in 3D-rendered image volumes (Fig. 2 D, arrows). To distinguish mechanical strain along actin cables from tension at focal adhesions, we excluded actin cables with zyxin localized solely at their ends. The percentage of zyxin-decorated actin cables increased markedly in 3-μm- compared with 8-μm-wide pores (Fig. 2 E). Consistent with enhanced actomyosin activity, the percentage of actin cables decorated with pMLC2 also increased in 3-μm- compared with 8-μm-wide pores (Fig. 2 F). Notably, SEPT9 presence on actin cables was similarly elevated in the narrower 3-μm-wide pores (Fig. 2 G), suggesting that SEPT9–actin association responds to the mechanical forces that promote confined migration. Consistent with this interpretation and previous work (Beadle et al., 2008; Ivkovic et al., 2012; Thomas et al., 2016), U2OS transmigration showed a greater dependence on myosin II activity at 3-μm- than 8-μm-wide pores (Fig. S3). Strikingly, SEPT9_i1 depletion resulted in a substantial increase of zyxin levels throughout the perinuclear cytoplasm in pores of all diameter sizes (Figs. 2, A–C and E; and S2, C–E). However, pMLC2 levels were not altered on actin cables (Fig. 2 F), suggesting that SEPT9 dampens tensile stress without affecting myosin II activation.

In addition to elevated zyxin, we observed that vertical actin cables were more bent in SEPT9-depleted cells (Fig. 2 H, arrows in insets 3 and 4), possibly due to the compressive and tensile stresses of enhanced contractility, as previously observed with reconstituted networks of nonsarcomeric actomyosin (Murrell and Gardel, 2012). We quantified bending of actin cables by measuring their linearity index (LI), which decreases with increasing curvature. The percentage of highly curved actin cables (LI < 0.5) was markedly higher in SEPT9-depleted cells (Fig. 2 I). Collectively, these data indicate that actomyosin forces and mechanical strain are substantially elevated in the absence of SEPT9_i1, suggesting that septins buffer the contractile forces of confined cell migration.

### Septins protect actin filaments from damage induced by myosin II hyperactivation

**Figure S4.**
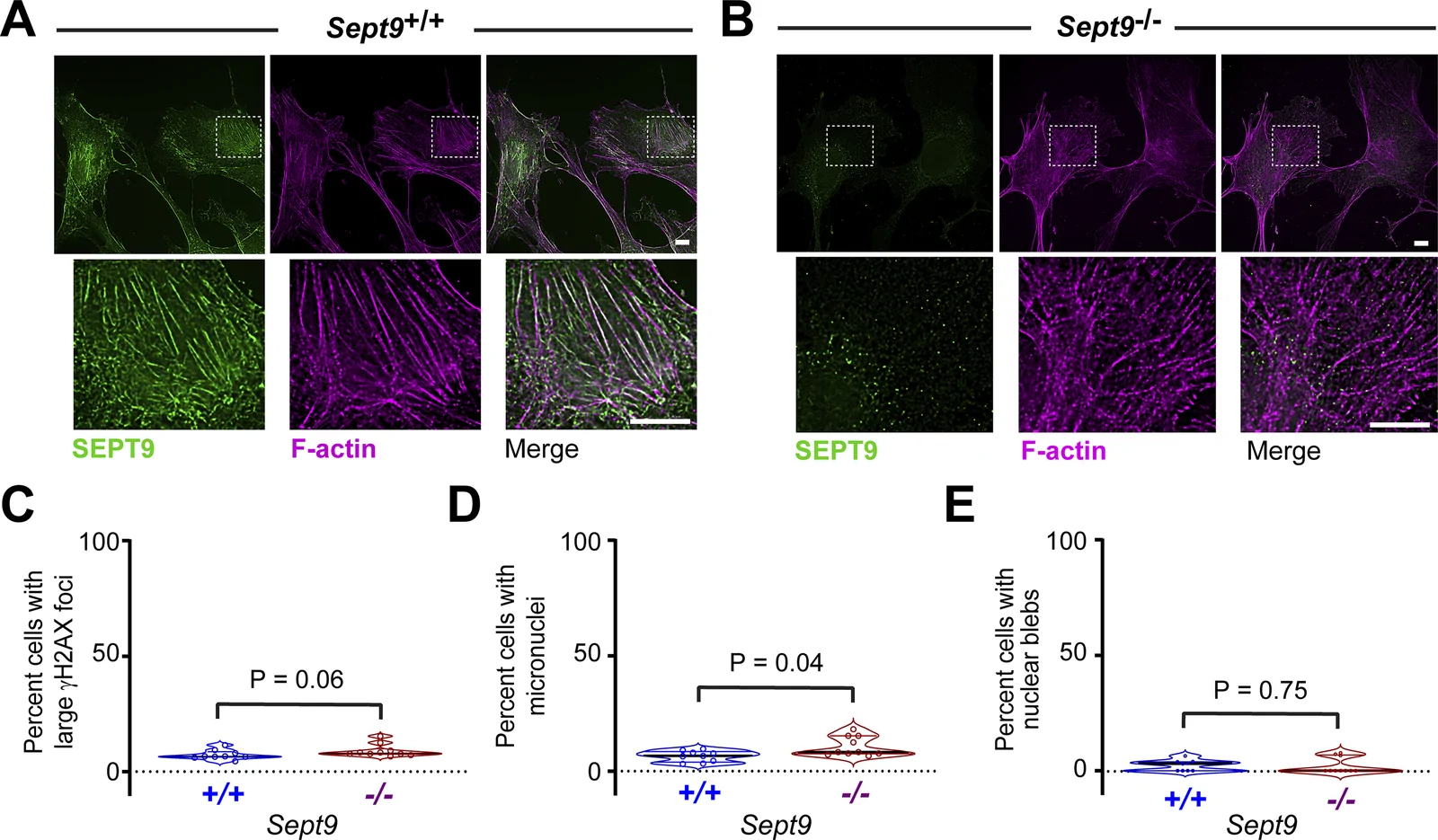
**SEPT9 expression and localization in *Sept9*** ^**+/+**^ **and *Sept9*** ^**−/−**^ **MEFs, and nuclear aberrations in the absence of confined migration. (A)** Wide-field microscopy images of *Sept9*^+/+^ MEFs on collagen type I ECM after staining for F-actin (phalloidin, magenta) and endogenous SEPT9 (green) with an antibody against all isoforms. Bottom panels show in higher magnification the outlined region with SEPT9 filaments colocalizing with actin stress fibers. Scale bars, 10 μm. **(B)** Wide-field microscopy images of *Sept9*^−/−^ MEFs on collagen type I ECM after staining for F-actin (phalloidin, magenta) and endogenous SEPT9 (green) with an antibody against all isoforms. Bottom panels show in higher magnification an outlined region with actin stress fibers, which lack SEPT9 filaments. The absence of SEPT9 expression is evident by the faint spotty background-level fluorescence signal. Scale bars, 10 μm. **(C)** Violin plots show the percentage of cells with large γH2AX foci after imaging apical regions of membranes with 3-μm-wide pores containing *Sept9*^+/+^ MEFs (*n* = 9 fields of view; 260 cells) and *Sept9*^−/−^ MEFs (*n* = 10 fields of view; 132 cells). Statistical comparisons were done with the Mann–Whitney U test. P = 0.06. **(D)** Violin plots show the percentage of cells with micronuclei after imaging apical regions of membranes with 3-μm-wide pores containing *Sept9*^+/+^ MEFs (*n* = 9 fields of view; 260 cells) and *Sept9*^−/−^ MEFs (*n* = 10 fields of view; 132 cells). Statistical comparisons were done with the Mann–Whitney U test. P = 0.04. **(E)** Violin plots show the percentage of cells with nuclear blebs after imaging apical regions of membranes with 3-μm-wide pores containing *Sept9*^+/+^ MEFs (*n* = 9 fields of view; 260 cells) and *Sept9*^−/−^ MEFs (*n* = 10 fields of view; 132 cells). Statistical comparisons were done with the Mann–Whitney U test. P = 0.75. All violin plots show the median (black line), and first and third quartiles (colored lines).

During confined 3D migration, a variety of forces—including frictional and hydrostatic—act on the nucleus and cytoskeleton, potentially damaging these structures through mechanical stress distinct from or additive to actomyosin-induced forces. We therefore sought to test whether septins buffer actomyosin forces using an orthogonal approach that relied on myosin II hyperactivation in the absence of additional mechanical constraints. We treated primary mouse embryonic fibroblasts (MEFs) from *Sept9*^−/−^ mice and littermate *Sept9*^*+/+*^ controls (Füchtbauer et al., 2011) (Fig. S4, A and B) with calyculin A, a potent inhibitor of protein phosphatase 1/2A that enhances phosphorylation of myosin regulatory light chain and consequently increases myosin II activity (Peterson et al., 2004). We cultured cells on 2D collagen-coated substrates to exclude actomyosin-mediated effects from confined migration.

**Figure 3.**
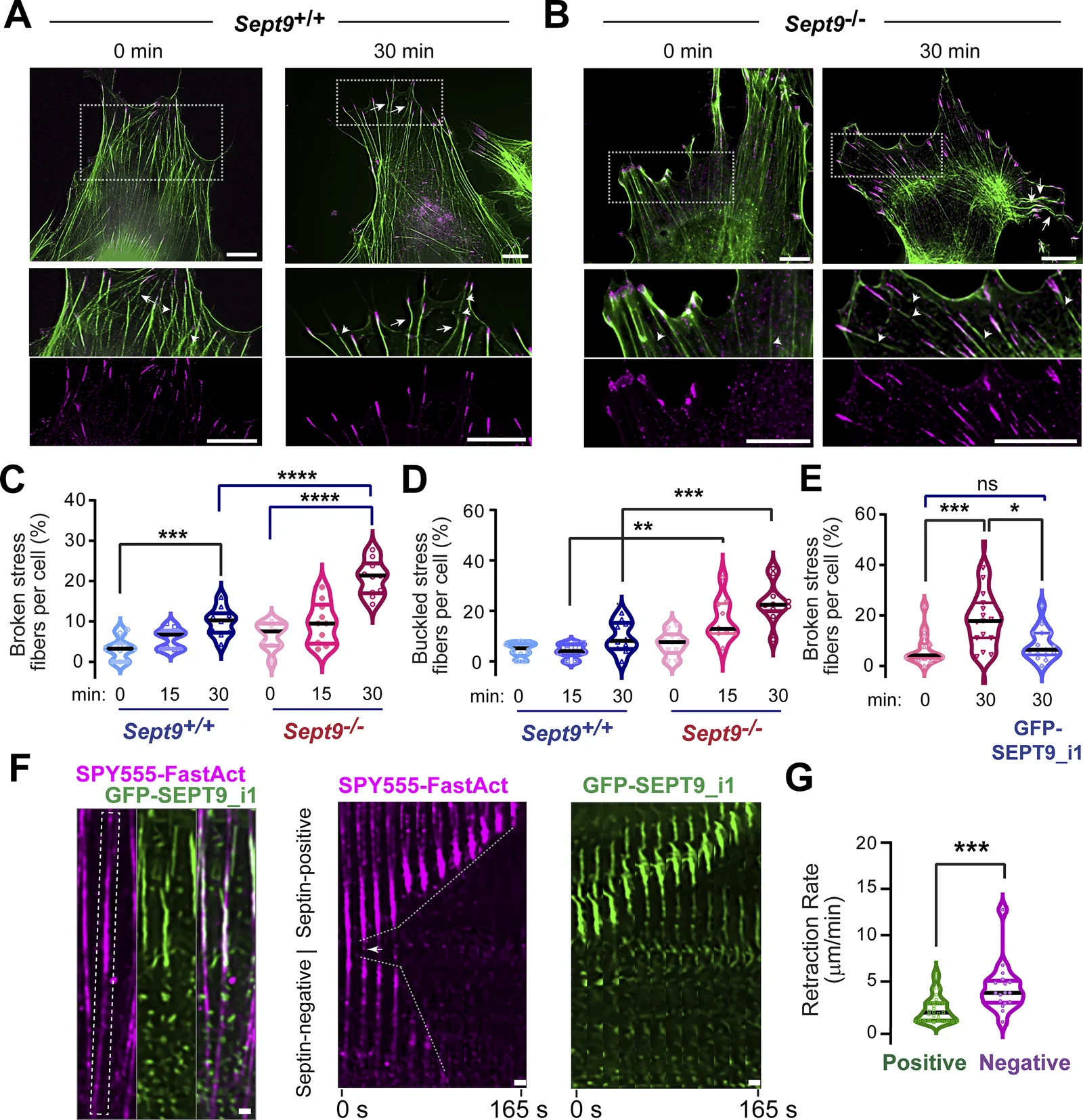
**Septins relieve actin stress fibers from tensile stress induced by myosin II hyperactivation. (A and B)** Wide-field microscopy images show F-actin (phalloidin, green) and paxillin (magenta) in MEFs from *Sept9*^+/+^ (A) and *Sept9*^−/−^ (B) mice, which were plated on collagen type I and treated with calyculin A (10 nM) for 30 min. Regions in dashed rectangles are shown in higher magnification below. Arrows and arrowheads point to breaks and buckles in actin stress fibers, respectively. Scale bars, 5 μm. **(C)** Violin plots show the percentage of actin stress fibers with breaks per cell after 0-min (*n* = 230–289 stress fibers; 10 cells), 15-min (*n* = 235–285 stress fibers; 10 cells), and 30-min (*n* = 239–255 stress fibers; 10 cells) treatment of *Sept9*^+/+^ and *Sept9*^−/−^ MEFs with calyculin A (10 nM). Statistical comparisons between the indicated groups were done with the Brown–Forsythe and Welch one-way ANOVA test with Dunnett’s T3 multiple comparisons test. ***, P < 0.001; ****, P < 0.0001. **(D)** Violin plots show the percentage of actin stress fibers with buckles per cell after 0-min (*n* = 230–289 stress fibers; 10 cells), 15-min (*n* = 235–285 stress fibers; 10 cells), and 30-min (*n* = 239–255 stress fibers; 10 cells) treatment of *Sept9*^+/+^ and *Sept9*^−/−^ MEFs with calyculin A (10 nM). Statistical comparisons between the indicated groups were done with the Brown–Forsythe and Welch one-way ANOVA test with Dunnett’s T3 multiple comparisons test. **, P < 0.01; ***, P < 0.001. **(E)** Violin plots show the percentage of stress fibers with breaks per cell in untreated *Sept9*^−/−^ MEFs (0 min; *n* = 467; 15 cells), and in *Sept9*^−/−^ MEFs with (*n* = 425; 15 cells) or without GFP-SEPT9_i1 (*n* = 376; 15 cells) after 30 min of calyculin A (10 nM) treatment. Statistical comparisons between the indicated pair groups were done with the Mann–Whitney U test. *, P < 0.05; ***, P < 0.001; ns, not significant. **(F)** Spinning-disk confocal microscopy image (left) and still frames (right) show a subcellular region of calyculin A (10 nM)–treated *Sept9*^−/−^ MEF cells, which expressed GFP-SEPT9_i1 (green) and were labeled with SPY555-FastAct (actin stress fibers, magenta). The dashed rectangle outlines the actin fiber shown in higher magnification in the still frames of kymograph. The arrow indicates the site of severing between the GFP-SEPT9_i1–coated and uncoated region of the stress fiber. Dashed lines mark the retraction trajectories of the GFP-SEPT9_i1–free and GFP-SEPT9_i1–coated actin segments. Scale bars, 1 μm. **(G)** Violin plots show the retraction rates (μm/min) for GFP-SEPT9_i1–free (*n* = 17) and GFP-SEPT9_i1–coated actin stress fibers (*n* = 29). Statistical comparison was done with the Mann–Whitney U test. ***, P < 0.001. All violin plots show the median (black line), and first and third quartiles (colored lines).

After treating MEFs with a low concentration of calyculin A (10 nM) for 15–30 min, we observed breaks in phalloidin-stained actin stress fibers (Fig. 3 A, arrowheads) proximal to peripheral focal adhesions, which are sites of higher mechanical tension. In *Sept9*^−/−^ MEFs, these breaks were more abundant (Fig. 3 B, arrowheads). Additionally, several actin stress fibers displayed a wavy appearance with multiple buckles (Fig. 3 B, arrows). Quantification of the percentage of stress fibers with phalloidin breaks revealed that actin severing increased progressively during calyculin A treatment between 0 and 30 min in both *Sept9*^*+/+*^ and *Sept9*^−/−^ MEFs (Fig. 3 C). After 30 min, the percentage of severed actin filaments was markedly higher in *Sept9*^−/−^ than *Sept9*^*+/+*^ MEFs (Fig. 3 C). Similarly, the percentage of buckled actin filaments was significantly elevated in *Sept9*^−/−^ compared with *Sept9*^+/+^ MEFs (Fig. 3 D). Enhanced buckling without significant severing after 15 min was consistent with buckling preceding severing in early stages of contraction, and severing occurring on actin segments of high curvature (Murrell and Gardel, 2012). Notably, the expression of human SEPT9_i1—an isoform that crosslinks actin filaments and is required for stress fiber organization (Dolat et al., 2014; Smith et al., 2015)—rescued the severing phenotype in *Sept9*^−/−^ MEFs, reducing the percentage of severed actin filaments to control levels after 30 min of calyculin A treatment (Fig. 3 E).

To further examine whether SEPT9_i1 reduces mechanical tension on actin stress fibers, we sought to image and analyze actin filament severing in real time in living cells. We posited that if SEPT9_i1 buffers tension on actin filaments, severing would occur preferentially on SEPT9_i1-free actin stress fibers. We tested this prediction by transfecting GFP-SEPT9_i1 into *Sept9*^*−/−*^ MEFs to generate a heterogeneous population of SEPT9_i1-coated and SEPT9_i1-free actin stress fibers, which were labeled with the vital dye SPY555-FastAct. Time-lapse imaging after calyculin A treatment revealed that 84% of severing events (*n* = 45) occurred on actin stress fiber segments lacking GFP-SEPT9_i1. Strikingly, on actin stress fibers that were partially coated with GFP-SEPT9_i1, the decorated segments retracted slower than the uncoated segments (Fig. 3, F and G; and Video 4). The differential retraction velocity indicates that SEPT9_i1-coated segments are under lower tension. Collectively, these results suggest that SEPT9_i1 reduces tension on actin filaments, protecting them from severing under hypercontractile conditions.

**Video 4.** **SEPT9_i1-coated actin filament segments retract more slowly than the septin-free segments after severing induced by myosin II hypercontractility.** Time-lapse spinning-disk confocal microscopy of *Sept9*^−/−^ MEFs expressing GFP-SEPT9_i1 (green) and labeled with SPY555-FastAct (magenta; F-actin). Cells were treated with calyculin A (10 nM) for 15 min prior to live imaging to induce myosin hypercontractility and actin severing events. Arrows indicate the site of actin severing and track the retracting end of the septin-free segment, which retracts faster than its septin-coated counterpart. Images were captured at 15-s intervals. Scale bar, 4 μm.

### Septins mitigate mechanical stress on the nuclear envelope from actomyosin contractility

**Figure 4.**
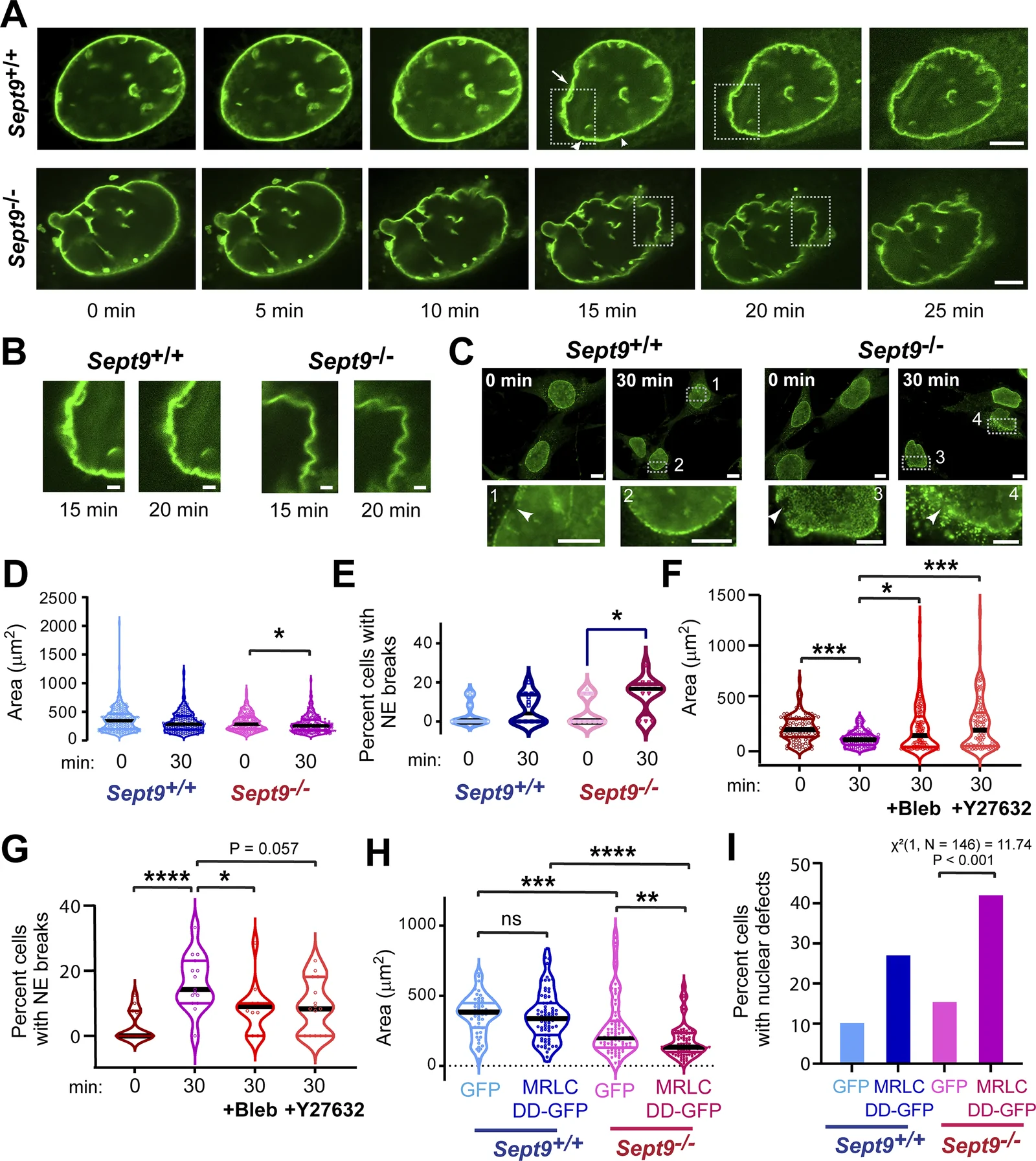
**Septins protect the nuclear envelope from the mechanical stress of actomyosin contraction. (A)** Still frames of SDCM images of live *Sept9*^+/+^ and *Sept9*^−/−^ MEFs expressing LAP2β-AcGFP1 (green) and treated with calyculin A (10 nM), which was administered 5 min prior to the start of time-lapse imaging. The arrow points to a flattened region of the circular nuclear envelope. Arrowheads point to nuclear envelope dimples. Scale bars, 5 μm. **(B)** SDCM images show in higher magnification the regions of the nuclear envelope outlined by the dashed lines in still frames of the time series above (A). Scale bars, 1 μm. **(C)** Wide-field microscopy images of *Sept9*^+/+^ and *Sept9*^*−/−*^ MEFs immunostained for nucleoporins after 0 and 30 min of treatment with calyculin A (10 nM). Numbered regions outlined by dashed lines are shown in higher magnification. Arrowheads point to sites of nuclear envelope rupture. Scale bars, 10 μm (5 μm in numbered panels). **(D)** Violin plots show nuclear surface areas in *Sept9*^+/+^ MEFs after 0 min (*n* = 243 cells) and 30 min (*n* = 210 cells) of calyculin A treatment, and in *Sept9*^−/−^ MEFs after 0 min (*n* = 202 cells) and 30 min (*n* = 208 cells) of calyculin A treatment. Statistical comparisons between the indicated groups were done with the Kruskal–Wallis ANOVA test (P < 0.0001) with Dunn’s correction for multiple comparisons test. *, P < 0.05. **(E)** Violin plots show the percentage of cells with ruptured nuclear envelopes in *Sept9*^+/+^ MEFs after 0 min (*n* = 12 fields of view, 53 cells) and 30 min (*n* = 12 fields of view, 38 cells) of calyculin A treatment, and in *Sept9*^−/−^ MEFs after 0 min (*n* = 12 fields of view, 50 cells) and 30 min (*n* = 12 fields of view, 50 cells) of calyculin A treatment. Statistical comparisons between the indicated groups were done with a nonparametric Kruskal–Wallis one-way ANOVA (P < 0.01) with Dunn’s multiple comparisons test. *, P < 0.05. **(F)** Violin plots show nuclear surface areas in *Sept9*^−/−^ MEFs after 0 min (*n* = 104 cells) and 30 min of calyculin A treatment in the absence (*n* = 100 cells) or presence of para-amino-blebbistatin (5 μM; *n* = 114 cells) and Y27632 (10 μM; *n* = 103). Statistical comparisons between the indicated groups were done with Kruskal–Wallis ANOVA test (P < 0.0001) with Dunn’s multiple comparisons test. ***, P < 0.001; *, P < 0.05. **(G)** Violin plots show the percentage of cells with ruptured nuclear envelopes in *Sept9*^−/−^ MEFs after 0 min (*n* = 15 fields of view; 104 cells) and 30 min (*n* = 15 fields of view; 100 cells) of calyculin A treatment in the absence (*n* = 15 fields of view; 100 cells) or presence of para-amino-blebbistatin (5 μM; *n* = 15 fields of view; 114 cells) and Y27632 (10 μM; *n* = 15 fields of view; 103 cells). Statistical comparisons between the indicated groups were done with the Kruskal–Wallis ANOVA test (P < 0.0001) with Dunn’s multiple comparisons test. ****, P < 0.0001; *, P < 0.05. **(H)** Violin plots show nuclear surface areas in MEFs expressing GFP (*n* = 52 *Sept9*^+/+^ MEFs; *n* = 94 *Sept9*^−/−^ MEFs) or MRLC-DD-GFP (*n* = 75 *Sept9*^+/+^ MEFs; *n* = 86 *Sept9*^−/−^ MEFs). Statistical comparisons between the indicated groups were done with the Kruskal–Wallis ANOVA test (P < 0.0001) with Dunn’s correction for multiple comparisons test. ns, not significant; **, P < 0.01; ***, P < 0.001; ****, P < 0.0001. **(I)** Bar graph shows the percentage of MEFs with nuclear defects (nuclear envelope ruptures, nuclear blebs) that express GFP (*n* = 45 *Sept9*^+/+^ MEFs; *n* = 76 *Sept9*^−/−^ MEFs) or MRLC-DD-GFP (*n* = 61 *Sept9*^+/+^ MEFs; *n* = 70 *Sept9*^−/−^ MEFs) for 36 h. Chi-square analysis with Yates’ continuity correction, and Fisher’s exact test and Bonferroni’s correction for multiple comparisons revealed no statistical significance between the *Sept9*^+/+^ MEF groups (5/45, 11.1% vs 17/61, 27.9%; χ^2^(1, *n* = 106) = 3.46, P = 0.063, Fisher’s P = 0.052; Bonferroni-adjusted P = 0.206–0.251) but a significant difference between *Sept9*^−/−^ MEFs (12/76, 15.8% vs 30/70, 42.9%; χ^2^(1, *N* = 146) = 11.74; Fisher’s test, P < 0.001; Bonferroni-adjusted P = 0.0018–0.0025). All violin plots show the median (black line), and first and third quartiles (colored lines). SDCM, spinning-disk confocal microscopy.

We reasoned that buffering of actomyosin tension by septins may extend to the nucleus, lessening the mechanical stress imposed on the nucleus during confined migration. To test if septins impact the forces transduced to the nuclear membrane by actomyosin contractility, we imaged the nuclear envelope (LAP2β-AcGFP1) during myosin II hyperactivation by calyculin A in *Sept9*^*+/+*^ and *Sept9*^−/−^ MEFs cultured on 2D collagen. After 15 min of calyculin A treatment, regions of the circular nuclear envelope began to flatten (Fig. 4 A, arrow) and crinkle forming small dimples (Fig. 4 A, arrowheads) in *Sept9*^*+/+*^ MEFs. In contrast, myosin II hyperactivation had an earlier and more dramatic effect in *Sept9*^−/−^ MEFs. The nuclear envelope began wrinkling after 10 min, and exhibited deep wrinkles and indentations assuming a jagged morphology after an additional 5–10 min (Fig. 4, A and B).

To further assess and quantify the impact of actomyosin hypercontractility and SEPT9 depletion on the nuclear envelope, we immunostained MEFs for nucleoporins. In agreement with real-time observations of the LAP2β-AcGFP1–labeled nuclear membrane, nuclear envelopes were less circular, possessing jagged and polygonal shapes after 30 min of calyculin treatment (Fig. 4 C). Several nuclei exhibited herniation and discontinuous nuclear rims, indicative of rupture (Fig. 4 C, arrowheads). Cytoplasmic nucleoporin foci, likely remnants of nuclear pore complexes released after rupture, were also seen at nuclear envelope gaps in *Sept9*^−/−^ MEFs (Fig. 4 C, inset 4 arrowhead). Quantification of nuclear areas revealed a reduction in calyculin-treated *Sept9*^*−/−*^ MEFs compared with untreated cells, whereas calyculin had no effect on *Sept9*^*+/+*^ cells relative to the *Sept9*^*−/−*^ group (Fig. 4 D); a standalone *t* test, however, showed a significant calyculin effect on *Sept9*^*+/+*^ cells, and the nuclear areas of these cells were also higher than those of calyculin-treated *Sept9*^*−/−*^ MEFs. Calyculin A also increased the percentage of cells with ruptured nuclear envelopes in *Sept9*^*−/−*^ MEFs, while *Sept9*^*+/+*^ were unaffected (Fig. 4 E). Attenuation of myosin II contractility by blebbistatin or the ROCK inhibitor Y27632 suppressed the effect of calyculin A on nuclear area (Fig. 4 F) and nuclear envelope integrity (Fig. 4 G) in *Sept9*^*−/−*^ MEFs. To independently corroborate that SEPT9 protects the nucleus from the forces of actomyosin contractility, we expressed the GFP-tagged constitutively active myosin regulatory light chain 1 T18D/S19D (MRLC-DD-GFP), which impacts nuclear morphology and integrity due to unrestrained contractility (Takaki et al., 2017). MRLC-DD-GFP reduced the nuclear area of *Sept9*^−/−^ MEFs and increased the percentage of cells with nuclear envelope rupture and blebs, while it had no statistically significant effect on *Sept9*^+/+^ cells (Figs. 4, H and I; and S5). Thus, loss of SEPT9 renders the nuclear envelope vulnerable to rupture, as unbuffered actomyosin forces exceed its mechanical tolerance.

**Figure S5.**
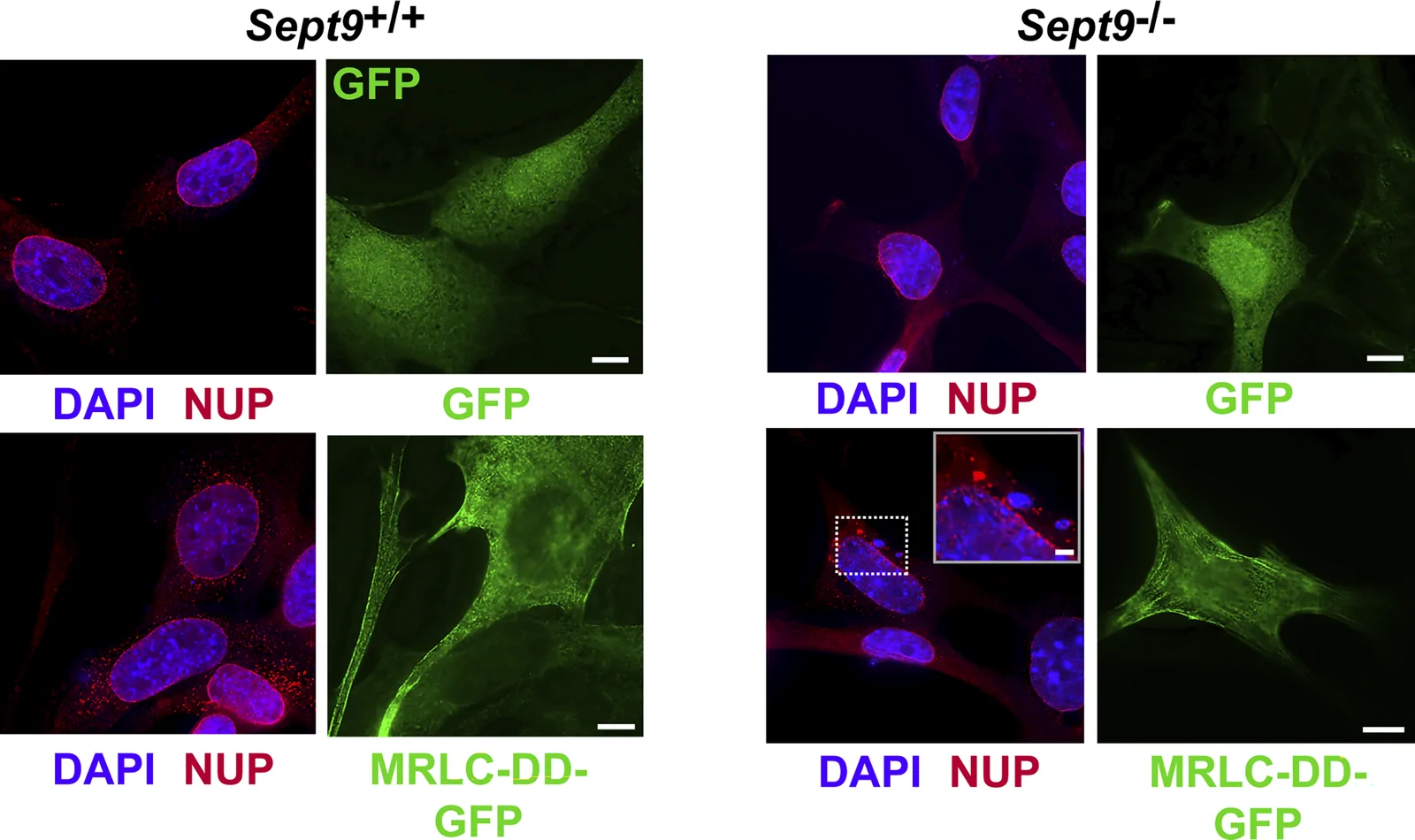
**Unrestricted myosin II contractility impairs nuclear integrity in *Sept9*** ^**−/−**^ **MEFs.** Images show wide-field microscopy images of *Sept9*^+/+^ and *Sept9*^−/−^ MEFs stained for nuclear DNA (DAPI, blue) and NUPs (red) after transfection with GFP or MRLC-DD-GFP (green) for 36 h. The inset shows a region with nuclear envelope rupture and nuclear bleb at the site of rupture. Scale bars, 10 μm (2 μm, inset). NUPs, nucleoporins.

### SEPT9_i1 protects the nucleus from genotoxic stress during confined migration, conferring survival advantage to breast cancer cells

The SEPT9 gene is amplified in murine models of breast cancer and human breast cancer cell lines (Connolly et al., 2011; Montagna et al., 2003; Scott et al., 2005). Importantly, the SEPT9_i1 isoform enhances cell migration (Connolly et al., 2011; Dolat et al., 2014; Farrugia et al., 2020; Hecht et al., 2024; Zeng et al., 2019) and induces the lung metastasis of mammary cells in a murine xenograft model (Zeng et al., 2019). Given that SEPT9_i1 expression alone was sufficient to reduce actomyosin tension in a SEPT9 pan-isoform knockout background (Fig. 3 E), we asked whether SEPT9_i1 amplification affects the mechanically induced genotoxic stress that cancer cells experience during confined migration (Kalukula et al., 2022; Maciejowski and Hatch, 2020; de Freitas Nader et al., 2021).

**Figure S6.**
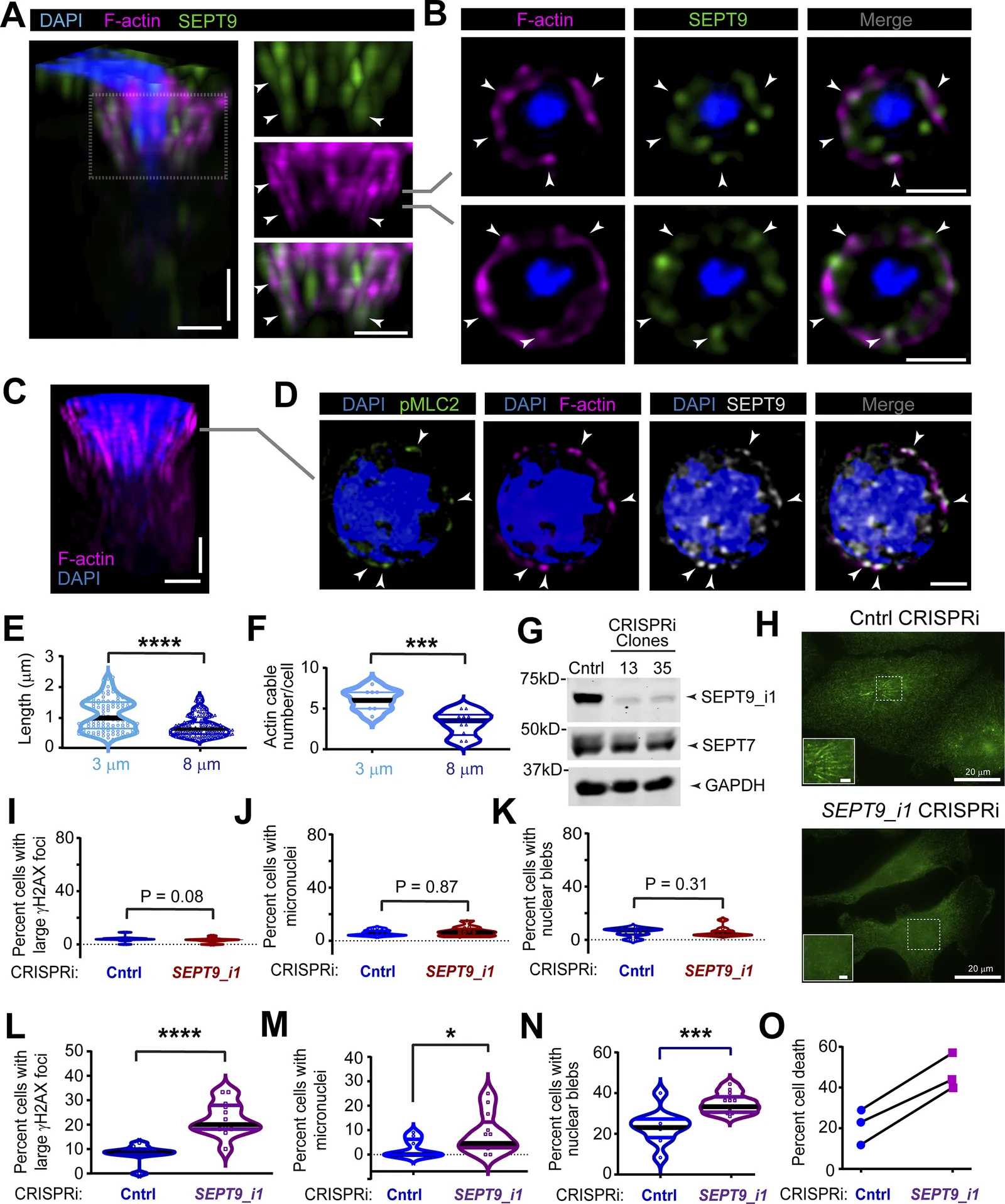
**SEPT9 expression and localization in MDA-MB-231 cells during confined migration, and effects of SEPT9_i1 depletion on genomic integrity and cell viability. (A)** Sagittal 3D volume view of an MDA-MB-231 cell region in a Matrigel/laminin-filled 3-μm-wide pore after staining for nucleus (DAPI, blue), F-actin (phalloidin, magenta), and SEPT9 (green). Images were acquired with 3D SoRa SDCM and rendered in 3D. Outlined regions are shown in higher magnification, and arrowheads point to SEPT9-decorated actin cables. Scale bars, 1 μm. **(B)** Images show single SoRa SDCM optical slices taken from the indicated depths of the 3-μm-wide pore (see lines in A). Arrowheads point to F-actin and SEPT9 colocalization. Scale bars, 1 μm. **(C)** Sagittal 3D volume view of an MDA-MB-231 cell region confined in a Matrigel-filled 3-μm-wide pore after staining for DAPI (blue) and F-actin (phalloidin, magenta). Images were acquired with 3D SoRa SDCM and rendered in 3D. Scale bars, 1 μm. **(D)** Images from a single SoRa SDCM optical slice taken from the indicated depth of the 3-μm-wide pore (see line in C), and stained for nucleus (DAPI, blue), F-actin (phalloidin, magenta), pMLC2 (green), and SEPT9 (grayscale). Arrowheads point to F-actin, SEPT9, and pMLC2 colocalization. Scale bar, 1 μm. **(E)** Violin plots of actin cable lengths in MDA-MB-231 cell regions confined in Matrigel/laminin-filled 3-μm (*n =* 106 filaments, 9 cells) and 8-μm (*n =* 131, 10 cells) pores. Statistical comparison was done with a Mann–Whitney U test. ****, P < 0.0001. **(F)** Violin plots of the number of actin cables with length >0.5 μm per MDA-MB-231 cell region confined in Matrigel/laminin-filled 3-μm (*n* = 9 cells) and 8-μm (*n =* 10 cells) pores. Statistical comparison was done with unpaired Student’s *t* test. ***, P < 0.001. **(G)** Western blots of lysates of MDA-MB-231 cell clones 13 (used below in L–O) and 35 (used in Fig. 5, A–F) after selection for stable expression of parental control and *SEPT9_i1*-targeting sgRNAs. Cell lysates were blotted with antibodies against the SEPT9_i1 isoform and SEPT7. **(H)** Wide-field microscopy images of MDA-MB-231 cells (clone 35) that stably express control and *SEPT9_i1* sgRNAs after immunostaining with an isoform-specific antibody against endogenous SEPT9_i1 (green). Insets show outlined regions in higher magnification. Scale bars, 20 μm (2 μm, insets). **(I)** Violin plots show the percentage of cells with large γH2AX foci after imaging apical regions of membranes with 3-μm-wide pores containing parental control (*n* = 13 fields of view, 315 cells) and SEPT9_i1-depleted MDA-MB-231 cells (clone 35; *n* = 11 fields of view, 325 cells) with large γH2AX foci. Statistical comparison was done with the Mann–Whitney U test. P = 0.08 (not significant). **(J)** Violin plots show the percentage of cells with micronuclei after imaging apical regions of membranes with 3-μm-wide pores containing parental control (*n* = 14 fields of view, 315 cells) and SEPT9_i1-depleted MDA-MB-231 cells (clone 35; *n* = 12 fields of view, 325 cells) with micronuclei. Statistical comparison was done with the Mann–Whitney U test. P = 0.87 (not significant). **(K)** Violin plots show the percentage of cells with nuclear blebs after imaging apical regions of polycarbonate membranes with 3-μm-wide pores containing parental control (*n* = 13 fields of view, 315 cells) and SEPT9_i1-depleted MDA-MB-231 cells (clone 35; *n* = 11 fields of view, 325 cells) with nuclear blebs. Statistical comparison was done with the Mann–Whitney U test. P = 0.31 (not significant). **(L)** Violin plots show the percentage of cells with large γH2AX foci after imaging basal regions of membranes with 3-μm-wide pores containing parental control (*n* = 11 fields of view, 121 cells) and SEPT9_i1-depleted MDA-MB-231 cells (clone 13; *n* = 13 fields of view, 147 cells) with large γH2AX foci. Statistical comparison was done with the Mann–Whitney U test. ****, P < 0.0001. **(M)** Violin plots show the percentage of cells with micronuclei after imaging basal regions of membranes with 3-μm-wide pores containing parental control (*n* = 11 fields of view, 121 cells) and SEPT9_i1-depleted MDA-MB-231 cells (clone 13; *n* = 13 fields of view, 147 cells) with micronuclei. Statistical comparison was done with the Mann–Whitney U test. *, P < 0.05. **(N)** Violin plots show the percentage of cells with nuclear blebs after imaging basal regions of membranes with 3-μm-wide pores containing parental control (*n* = 11 fields of view, 121 cells) and SEPT9_i1-depleted MDA-MB-231 cells (clone 13; *n* = 13 fields of view, 147 cells) with nuclear blebs. Statistical comparison was done with an unpaired *t* test with Welch’s correction. ***, P < 0.001. **(O)** Percentage of trypan blue–positive cells after trypsinization of parental control (*n* = 4,185, 3,400, 3,660) and SEPT9_i1-depleted MDA-MB-231 cells (clone 13; *n* = 5,500, 2,620, 4,710) from the basal surfaces of membranes with 3-μm-wide pores, following 36 h of apicobasal transmigration. Results from three independent experiments are shown. All violin plots show the median (black line), and first and third quartiles (colored lines). SDCM, spinning-disk confocal microscopy. Source data are available for this figure: SourceData FS6.

We first examined whether the highly invasive human breast cancer cell line MDA-MB-231 (Minn et al., 2005), which harbors multiple SEPT9 gene copies (Connolly et al., 2011), contains a perinuclear septoactomyosin cable arrangement similar to U2OS cells during confined migration. MDA-MB-231 cells possessed actin cables that surrounded the nucleus within narrow 3-μm-wide pores, aligning with SEPT9 along the apicobasal axis of the pore (Fig. S6, A and B, arrowheads, and Video 5). Similar to U2OS cells, perinuclear regions had approximately twofold (1.85 ± 0.25; *n* = 10 cells) more actin cables than nuclear-free areas. Indicative of contractile activity, pMLC2 localized to several actin cables along with SEPT9 (Fig. S6, C and D, arrowheads). In spite of the reduced volume of the pore-occupying cytoplasm in 3-μm- compared with 8-μm-wide pores, the length and number of actin cables increased in 3-μm-wide pores (Fig. S6, E and F), potentially an adaptive response to the higher forces necessary to overcome increasing physical constraints.

**Video 5.** **Septin-associated actomyosin cables surround the nucleus in a pore-occupying region of an MDA-MB-231 cell.** Super-resolution SoRa spinning-disk confocal microscopy of an MDA-MB-231 cell region within a 3-μm-wide pore, showing F-actin (phalloidin, magenta), SEPT9 (grayscale), and pMLC2 (green) after 3D volume rendering. The image initially rotates 90° around the horizontal xy axis for viewing in 3D sideways from an oblique angle. The fluorescence signals are sequentially displayed: pMLC2 and SEPT9 signals fade first simultaneously, revealing actin cables alone (magenta). Subsequently, SEPT9 signal is restored prior to fading, and overlaying of pMLC2 onto F-actin with a 180° rotation around the vertical axis of the pore. The beaded appearance of some actin cables is due to sub-Nyquist sampling, which was necessary to offset photobleaching during acquisition of a large image z-stack. Scale bar, ∼2 μm.

**Figure 5.**
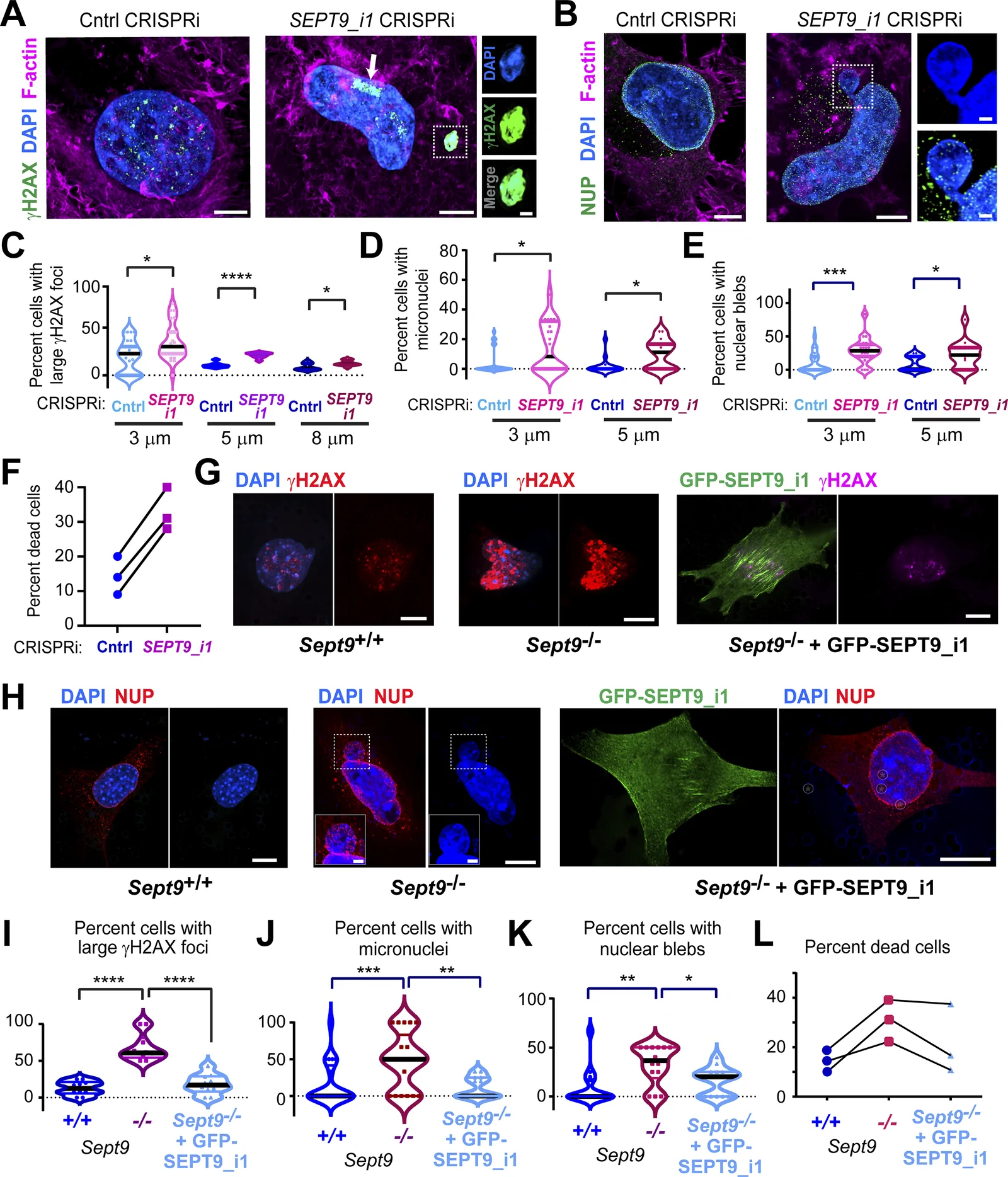
**SEPT9_i1 protects nuclei from mechanical damage during confined migration, suppressing DNA damage, nuclear defects associated with genomic instability, and cell death. (A)** SoRa SDCM images of nuclei (DAPI, blue), γH2AX (green), and F-actin (phalloidin, magenta) in parental control and SEPT9_i1-depleted MDA-MB-231 cells, taken from the basal side of membranes with 3-μm-wide pores after 36 h of apicobasal transmigration. The arrow points to a large nuclear region with γH2AX accumulation. The inset shows a γH2AX-enriched micronucleus in higher magnification. Scale bars, 5 μm (1 μm in inset). **(B)** SDCM images of nuclei (DAPI, blue), NUPs (green), and F-actin (phalloidin, magenta) in parental control and SEPT9_i1-depleted MDA-MB-231 cells, taken from the basal side of membranes with 3-μm-wide pores after 36 h of apicobasal transmigration. The inset shows in higher magnification a region of nuclear blebbing, where nuclear DNA protrudes from a site of nuclear envelope rupture. Scale bars, 5 μm (1 μm in inset). **(C)** Violin plots show the percentage of parental control and SEPT9_i1-depleted MDA-MB-231 cells with large γH2AX foci on basal membrane regions with 3-μm-wide (*n* = 27 fields of view; 83–96 cells), 5-μm-wide (*n* = 9 fields of view; 74–89 cells), and 8-μm-wide (*n* = 9 fields of view; 80–106 cells) pores. Statistical comparisons between the indicated pair groups were done with the Mann–Whitney U test. *, P < 0.05; ****, P < 0.0001. **(D)** Violin plots show the percentage of parental control and SEPT9_i1-depleted MDA-MB-231 cells with micronuclei on basal membrane regions with 3-μm-wide (*n* = 20–22 fields of view; 88–95 cells) and 5-μm-wide (*n* = 15 fields of view; 80–85 cells) pores. Statistical comparisons between the indicated pair groups were done with the Mann–Whitney U test. *, P < 0.05. **(E)** Violin plots show the percentage of parental control and SEPT9_i1-depleted MDA-MB-231 cells with nuclear blebs on basal membrane regions with 3-μm (*n* = 20–22 fields of view; 88–95 cells) and 5-μm (*n* = 15 fields of view, *n* = 80–85 cells) pores. Statistical comparisons between the indicated pair groups were done with the Mann–Whitney U test. *, P < 0.05; ***, P < 0.001. **(F)** Percentage of trypan blue–positive cells after trypsinization of parental control (*n* = 5,230, 3,660, 2,620) and SEPT9_i1-depleted MDA-MB-231 cells (*n* = 4,190, 3,140, 2,090) from the basal surfaces of membranes with 3-μm-wide pores, following 36 h of apicobasal transmigration. Results from three independent experiments are shown. **(G)** SDCM images of nuclei (DAPI, blue), γH2AX (red), and GFP-SEPT9_i1 (green) in *Sept9*^+/+^ and/or *Sept9*^−/−^ MEFs, taken from the basal side of membranes with 3-μm-wide pores after 36 h of apicobasal transmigration. Scale bars, 10 μm. **(H)** SDCM images of nuclei (DAPI, blue), NUPs (red), and GFP-SEPT9_i1 (green) in *Sept9*^+/+^ and/or *Sept9*^−/−^ MEF cells, taken from the basal side of membranes with 3-μm-wide pores after 36 h of apicobasal transmigration. Scale bars, 10 μm (2 μm, inset). **(I)** Violin plots show the percentage of cells with large γH2AX foci after imaging basal regions of membranes with 3-μm-wide pores containing *Sept9*^+/+^ MEFs (*n* = 10 fields of view; 85 cells) and *Sept9*^−/−^ MEFs with (*n* = 10 fields of view; 73 cells) and without GFP-SEPT9_i1 (*n* = 10 fields of view; 83 cells). Statistical comparisons between the indicated groups were done with the Brown–Forsythe and Welch ANOVA test with Dunnett’s T3 multiple comparisons test. ****, P < 0.0001. **(J)** Violin plots show the percentage of cells with micronuclei after imaging basal regions of membranes with 3-μm-wide pores containing *Sept9*^+/+^ MEFs (*n* = 21 fields of view; 48 cells) and *Sept9*^−/−^ MEFs with (*n* = 21 fields of view; 35 cells) and without GFP-SEPT9_i1 (*n* = 13 fields of view; 63 cells). Statistical comparisons between the indicated groups were done with the Brown–Forsythe and Welch ANOVA test with Dunnett’s T3 multiple comparisons test. **, P < 0.01; ***, P < 0.001. **(K)** Violin plots show the percentage of cells with nuclear blebs after imaging basal regions of membranes with 3-μm-wide pores containing *Sept9*^+/+^ MEFs (*n* = 14 fields of view; 51 cells) and *Sept9*^−/−^ MEFs with (*n* = 14 fields of view; 45 cells) and without GFP-SEPT9_i1 (*n* = 14 fields of view; 51 cells). Statistical comparisons between the indicated groups were done with the Brown–Forsythe and Welch ANOVA test with Dunnett’s T3 multiple comparisons test. **, P < 0.01; *, P < 0.05. **(L)** Percentage of trypan blue–positive cells after trypsinization of *Sept9*^+/+^ (*n =* 4,115, 2,939, 4,700), *Sept9*^−/−^ (*n =* 6,760, 5,580, 2,645), and *Sept9*^−/−^ MEFs transfected with GFP-SEPT9_i1 (*n =* 2,175, 1,764, 7,055) for 24 h prior to confined migration for 36 h through 3-μm-wide pores. Results from three independent experiments are shown. Note that transfection efficiency was ∼40%, and cell death was measured in the entire population of *Sept9*^−/−^ MEFs and not strictly in the cells expressing GFP-SEPT9_i1. All violin plots show the median (black line), and first and third quartiles (colored lines). SDCM, spinning-disk confocal microscopy; NUPs, nucleoporins.

We asked whether MDA-MB-231 cells are prone to increasing DNA damage after migration through pores of decreasing diameters, which is predicted to scale with increasing forces, and whether SEPT9_i1 expression affects the damage, which can be induced by actomyosin contractility during confined migration (Hatch and Hetzer, 2016; Takaki et al., 2017; Xia et al., 2019). Using CRISPR, we generated stable SEPT9_i1-depleted MDA-MB-231 cells (Fig. S6, G and H). We assessed DNA lesions in cells that had fully emerged onto the basal membrane surface after 36 h of EGF-guided chemotactic migration. Of note, the basal side was coated with the same ECM type that filled the pores and coated the apical side. Immunostaining for γH2AX, a marker of DNA double-strand breaks (DDSBs), revealed a marked increase in the percentage of control cells with large γH2AX foci among those that emerged from 3-μm pores compared with 5-μm- and 8-μm-wide pores (Fig. 5, A and C). Notably, DNA damage increased in SEPT9_i1-depleted MDA-MB-231 compared with controls after confined migration through 8-, 5-, or 3-μm-wide pores (Fig. 5 C).

We next analyzed cells for micronuclei and nuclear blebs, biomarkers of genomic instability resulting from excessive DNA damage and nuclear rupture (Mammel and Hatch, 2022). We stained cells for nucleoporins and DAPI to visualize DNA, quantifying micronuclei as cytoplasmic islands of DNA lacking nucleoporins (Fig. 5 A, inset) and nuclear blebs as nuclei with DNA bulging out through discontinuities in the nuclear envelope (Fig. 5 B, inset). We focused this analysis on cells that emerged from narrower 3- to 5-μm-wide pores, which induce higher mechanical stress. SEPT9_i1-depleted cells showed a statistically significant increase in both micronuclei and nuclear blebs after migration through 3- and 5-μm pores (Fig. 5, D and E). Notably, the percentage of cells with nuclear blebs was markedly higher than those with micronuclei, with nearly 25% of SEPT9_i1-depleted cells containing nuclear blebs (Fig. 5 E). As micronuclei typically form after mitosis following DNA damage (Harding et al., 2017), the higher prevalence of nuclear blebs may reflect a greater proportion of cells that had not yet undergone cell division after nuclear rupture. These effects were the consequence of confined migration as SEPT9-depleted cells did not exhibit increased DNA damage, micronuclei, or nuclear blebs on the apical side of the porous membranes (Fig. S6, I–K).

To examine whether cells that emerge from confined migration are less viable, we assayed for cell death. We harvested cells adhered to the basal surface of the polymeric membranes following confined migration through 3-μm-wide pores and labeled them with trypan blue to distinguish live from dead cells. We found that 25–40% of SEPT9_i1-depleted cells were dead, representing a two- to threefold increase compared with control MDA-MB-231 cells (Fig. 5 F). Similar results were obtained independently using a different SEPT9_i1-depleted MDA-MB-231 clone, which also exhibited increased DNA damage, micronuclei, nuclear blebs, and cell death after confined migration (Fig. S6, L–O).

We next asked whether the oncogenic SEPT9_i1 isoform alone can limit the genotoxic mechanical damage, which is caused by confined migration. Because SEPT9_i1 expression rescued actin stress fiber damage in *Sept9*^*−/−*^ MEFs under hypercontractile conditions induced by myosin II hyperactivation, restoring actin stress fiber integrity to control levels (Fig. 3 E), we tested whether SEPT9_i1 expression can suppress genotoxic damage and cell death during confined migration in MEF cells. Following confined migration through 3-μm pores, the percentage of *Sept9*^*−/−*^ MEFs with DDSBs, micronuclei, and nuclear blebs was substantially elevated compared with *Sept9*^*+/+*^ MEFs (Fig. 5, G and H). Approximately 50% of *Sept9*^*−/−*^ MEFs exhibited DNA damage and micronuclei (Fig. 5, I and J), while ∼30% contained nuclear blebs (Fig. 5 K). Prior to transmigration, however, *Sept9*^*−/−*^ MEFs resembled the matched controls in low levels of DDSBs, micronuclei, and nuclear blebs—<10% of total cells (Fig. S4, C–E). In addition to the nuclear abnormalities, cell death also increased in *Sept9*^*−/−*^ MEFs following migration (Fig. 5 L). The expression of GFP-SEPT9_i1 in *Sept9*^*−/−*^ MEFs rescued DNA damage and micronuclei, restoring the percentage of affected cells to *Sept9*^*+/+*^ levels (Fig. 5, I and J), and also reduced nuclear blebs and cell death (Fig. 5, K and L). Taken together, these results show that SEPT9_i1 protects cells from the consequences of confined migration—DNA damage, genomic instability, and cell death—providing a survival advantage. Thus, the function of SEPT9_i1 in buffering actomyosin forces extends to protecting the nuclear and genomic integrity.

### *SEPT9* amplification associates with lower thresholds of genomic alteration in aggressive tumors

Metastatic cancers are characterized by elevated genomic instability, which not only promotes tumor evolution but also imposes constrains on cancer cell viability (Andor et al., 2017; Hosea et al., 2024; Zhivotovsky and Kroemer, 2004). Excessive genomic alterations compromise cell fitness and can trigger apoptosis and immunogenic cell death (Andor et al., 2017; Zhivotovsky and Kroemer, 2004). During metastatic dissemination, which involves confined migration, tumor cells are subjected to recurrent nuclear and DNA damage during confined migration, increasing the likelihood that highly altered genomes exceed tolerable limits of genomic instability (Shah et al., 2017). This points to cellular mechanisms that permit continued tumor survival by limiting catastrophic genomic damage as evidenced by the low survival of tumors with excessive genomic alterations, the persistence of early (truncal) oncogenic mutations throughout cancer progression, and the presence of islands of relative genomic stability in metastatic tumor subclones (Andor et al., 2017; Bowland et al., 2024; Levine et al., 2019).

Our results suggest that SEPT9_i1 protects cancer cells from the genotoxic mechanical stress of confined migration. *SEPT9* amplification may therefore enable cancer cells to tolerate substantial genomic instability within a permissive threshold, potentially without preventing the accumulation of smaller alterations, which might be beneficial for tumor progression. To assess whether this model is reflected in clinical outcomes, we examined genomic data from cancer patients.

**Figure 6.**
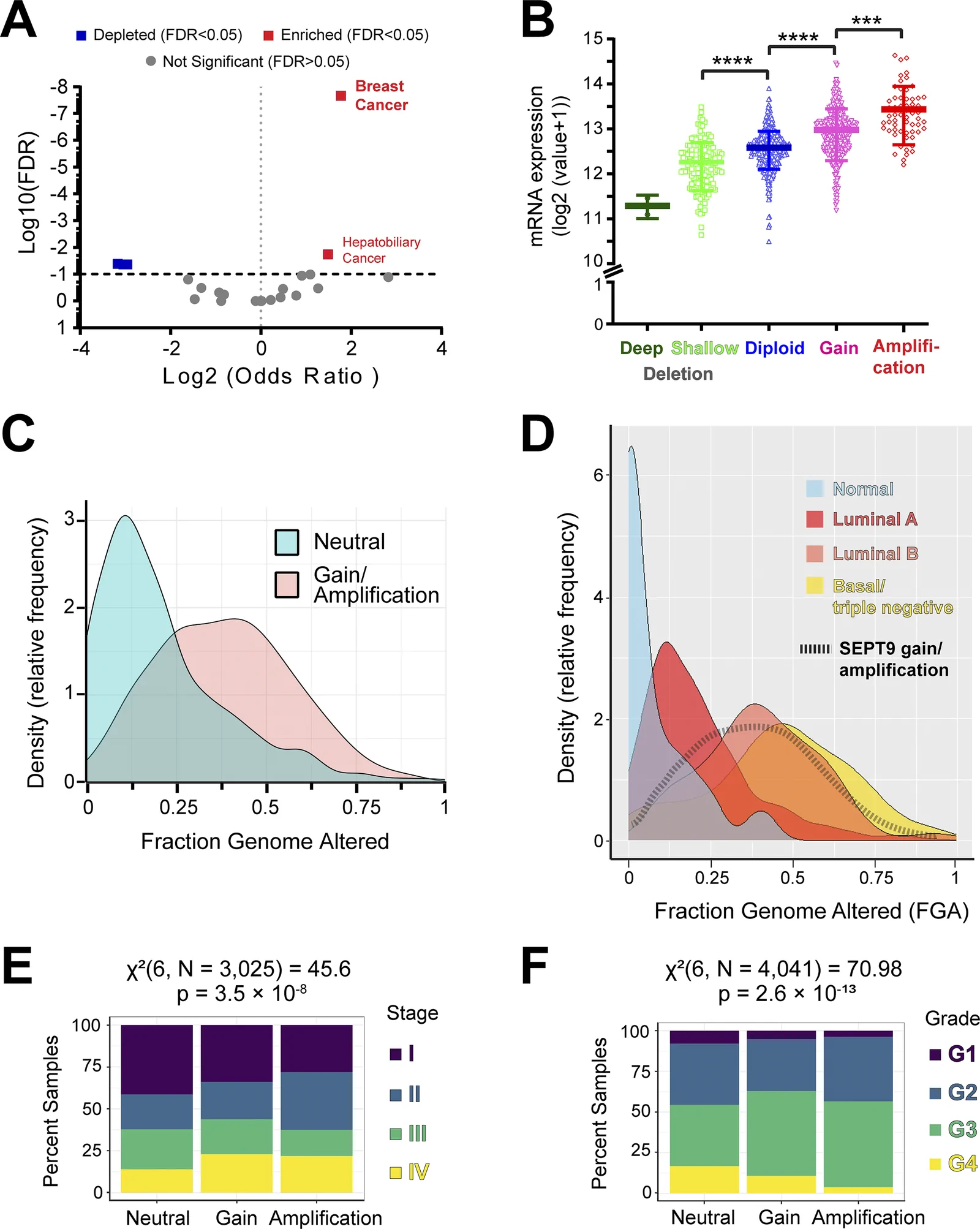
***SEPT9* amplification associates with suppression of excessive genomic alteration. (A)** Volcano plot showing log2 odds ratios for *SEPT9* amplification (positive values) and deletion (negative values) across tumor types, plotted against statistical significance (log10 FDR). Tumor types with FDR <0.05 are highlighted. Breast cancer shows the strongest enrichment for *SEPT9* amplification, while more modest enrichment is observed in hepatobiliary cancer. Several tumor types including (blue squares) glioblastoma, head and neck cancer, thyroid cancer, and renal clear cell carcinoma exhibit significant depletion of *SEPT*9 copy number. Data were derived from TCGA. **(B)** SEPT9 mRNA expression (log2 [value + 1]) is shown for breast tumors stratified by *SEPT9* GISTIC copy-number state: deep (*n* = 2) and shallow (*n* = 153) deletion, diploid (*n* = 447), gain (*n* = 299), and amplification (*n* = 59). Statistical analysis was assessed using the Kruskal–Wallis test (P < 0.0001), followed by Dunn’s multiple comparisons test (****, P <0.0001; ***, P < 0.001). **(C)** Kernel density plot showing the relative frequency of FGA values in breast tumors with copy-neutral *SEPT9* (cyan; *n* = 517) and tumors with *SEPT9* copy-number gain or amplification (pink; *n* = 403). **(D)** Kernel density plots showing the distribution of FGA values in normal breast tissue (*n* = 36), luminal A breast cancer (*n* = 496), luminal B breast cancer (*n* = 197), basal/triple-negative breast cancer (*n* = 171), and all breast tumors harboring *SEPT9* copy-number gain or amplification (*n* = 403). **(E)** Stacked bar plot showing the distribution of simplified tumor stages (I–IV) across *SEPT9* copy-number states (neutral, gain, amplified) in primary tumors from TCGA. Proportions are calculated within each *SEPT9* copy-number category. *SEPT9* gain and amplification are progressively enriched among higher stage tumors. Statistical significance was assessed using a chi-square test (χ^2^(6, *N* = 3,025) = 45.64, P = 3.5 × 10^−8^). **(F)** Stacked bar plot depicting the distribution of histologic tumor grades (G1–G4) stratified by *SEPT9* copy-number status across primary tumors. Only samples with interpretable grade annotations (G1–G4) were included. *SEPT9* gain and amplification are associated with increased proportions of higher grade tumors. Statistical significance was evaluated using a chi-square test (χ^2^(6, *N* = 4,041) = 70.98, P = 2.6 × 10^−13^). G1, well differentiated (low grade); G2, moderately differentiated (intermediate grade); G3, poorly differentiated (high grade); G4, undifferentiated (high grade).

Systematic analysis of *SEPT9* gene copy-number alterations in large clinicogenomic cohorts is lacking. To assess tumor-type specificity of *SEPT9* copy-number alterations, we examined *SEPT9* amplification frequency across all TCGA primary tumors with available copy-number data (*n* = 11,245). *SEPT9* amplification (>5 copies) was most frequent in invasive breast, renal, and hepatic cancers, occurring in ∼25% of cases within each cohort. To formally evaluate tumor-type enrichment of high-level *SEPT9* amplification, we performed enrichment analyses restricted to amplification-only events in tumor types with sufficient sample size (*N* ≥ 20). Breast cancer emerged as the most significantly enriched tumor type for *SEPT9* amplification (odds ratio = 3.42, BH-FDR = 2.16 × 10^−8^), with more modest enrichment observed in hepatobiliary cancers (odds ratio = 2.80, BH-FDR = 0.018) (Fig. 6 A).

Because gene copy-number alterations do not necessarily imply functional relevance, we next examined *SEPT9* mRNA expression in breast cancer tumors stratified by the *SEPT9* gene copy-number status. Mean *SEPT9* mRNA expression increased in a stepwise manner with increasing *SEPT9* gene copy number, including copy-number gain and amplification (Fig. 6 B). This concordance between gene copy-number increase and transcriptional upregulation is consistent with dosage-dependent regulation and mirrors patterns observed for established oncogenes in breast cancer, such as *ERBB2* (*HER2*) (Shahrouzi et al., 2024; Shao et al., 2019).

To determine whether *SEPT9* gene copy gain and amplification simply reflect elevated chromosomal instability, we examined the distribution of fractional genome altered (FGA) across tumors stratified by *SEPT9* copy-number status in breast cancers. The relative frequency of *SEPT9* gain and amplification varied across the full spectrum of FGA values, exhibiting a nonmonotonic distribution that peaked at intermediate levels of chromosomal instability (FGA ∼0.25–0.5) and declined at higher FGA values (0.5–0.7) (Fig. 6 C). Exclusion of the *SEPT9*-containing chromosome 17 from both the altered-genome numerator and the evaluated-genome denominator did not alter this distribution. Median FGA was 0.164 (*n* = 517) and 0.393 (*n* = 403) in *SEPT9* copy neural and gain/amplification tumors, respectively, compared with 0.162 and 0.399 in whole-genome analysis. Notably, the FGA range over which *SEPT9* gain and amplification were most frequent overlaps with that of aggressive breast cancer subtypes with higher recurrence rates and adverse clinical outcomes, including luminal B and basal/triple-negative tumors (Fig. 6 D). Interestingly, however, cells with FGAs over 0.6 were less frequent in breast cancers with *SEPT9* gain and amplification than in basal/triple-negative tumors (Fig. 6 D). A pan-cancer analysis also revealed a strong relationship between *SEPT9* copy-number state (neutral, gain, amplification) and tumor stage and grade, with *SEPT9* gain and amplification progressively enriched in tumors with higher stage and histologic grades (Fig. 6, E and F). These data indicate that *SEPT9* gene amplification associates with aggressive tumors with intermediate-to-high levels of genome alteration, which have a survival advantage over tumors with low or excessive levels of chromosomal instability.

If *SEPT9* amplification enables tumor cells to tolerate genomic instability, a key question is whether this tolerance involves selective constraints that limit the accumulation of larger scale genomic changes while allowing lower scale alterations. We examined this by analyzing the relationship between *SEPT9* copy-number status and tumor mutation burden (TMB), a measure of smaller scale genomic alterations from single nucleotide and small insertion–deletion events.

In contrast to FGA, *SEPT9* gain or amplification was not associated with reduced mutation burden. In tumor types enriched for *SEPT9* copy-number alterations, tumors harboring *SEPT9* gain or amplification exhibited comparable or modestly higher log-transformed TMB compared with copy-neutral tumors, whereas no significant difference was observed in pan-cancer analyses. In addition, *SEPT9* gain or amplification was not associated with a reduced probability of hypermutation, which was defined as tumors in the upper tail of the TMB distribution within each cancer type (top 20%). In tumor types enriched for *SEPT9* copy-number alterations, hypermutation was observed at comparable or higher frequencies in *SEPT9*-altered tumors compared with copy-neutral tumors (odds ratio = 1.65, FDR <1 × 10^−4^), whereas no enrichment or depletion was observed in pan-cancer analyses (Table S1).

**Figure 7.**
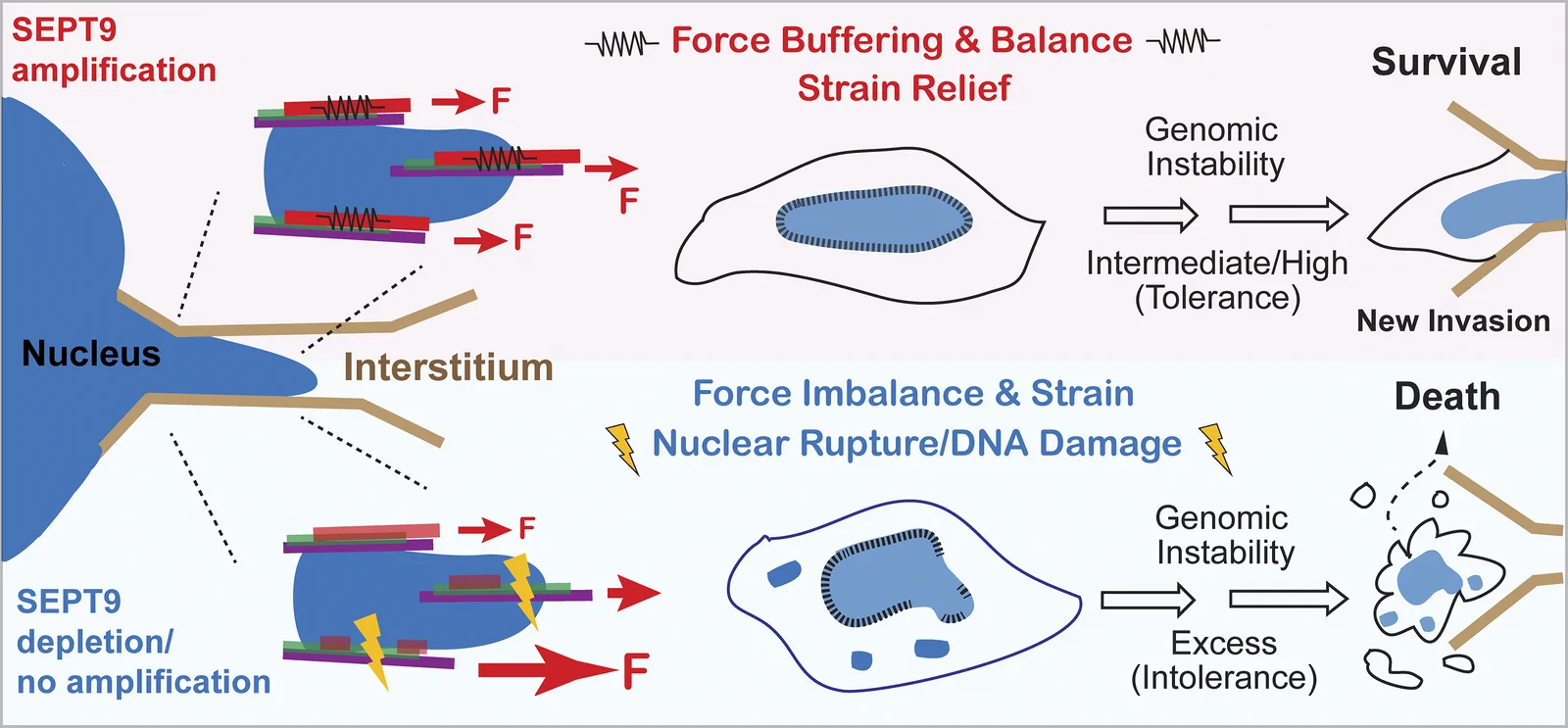
**Working model: *SEPT9* amplification buffers mechanical forces during confined migration to maintain a permissive window of genomic instability.** The schematic illustrates the proposed role of *SEPT9* amplification in buffering actomyosin-generated forces during confined migration to maintain genomic instability within tolerable limits. We posit that through this mechanism, *SEPT9* amplification enables metastatic tumor cells to navigate a narrow survival window, avoiding lethal genomic catastrophe, which ultimately promotes metastatic colonization and diminishes patient survival. In tumor cells with lower levels of SEPT9 expression, the nucleus is more prone to mechanical damage during confined migration, leading to large-scale genomic alterations that surpass a threshold of cell tolerance and result in cell death.

Collectively, these results indicate that *SEPT9* gain and amplification are not simply a byproduct of extreme genome-wide chromosomal instability and do not appear to associate with lower mutational burden. Instead, *SEPT9* amplification may enable tumor cells to tolerate substantial chromosomal instability without crossing lethal thresholds of genomic damage, and thereby provide a selective advantage for aggressive tumors in certain metastatic niches. In this model (Fig. 7), *SEPT9* protects the nucleus from catastrophic damage during confined migration while permitting continued accumulation of smaller scale genetic alterations that can contribute to tumor heterogeneity, clonal evolution, and reduced clinical outcome (Tubbs and Nussenzweig, 2017).

## Discussion

Septins comprise a distinct cytoskeletal network—often referred to as the fourth component of the cytoskeleton alongside actin, microtubules, and intermediate filaments (Mostowy and Cossart, 2012). While septins have been reported to influence plasma membrane rigidity and the nucleocytoplasmic transport of mechanosensitive transcription factors (Calvo et al., 2015; Gilden and Krummel, 2010), they have been largely overlooked as a mechanical element of the cell. Here, we reveal a previously unknown septin function in buffering actomyosin forces, and thereby protecting both actin cytoskeleton and the nucleus from mechanical stress and damage.

Septins are neither evolutionarily nor structurally related to intermediate filaments, which are flexible and stretchable structures that dissipate mechanical stress through deformation and reorganization (Guo et al., 2025; Swoger et al., 2025). This raises the question of how septins can buffer mechanical tension. Intriguingly, yeast septin filaments have persistence lengths of ∼1–2 μm, approaching those of the pliable intermediate filaments (0.2–1 μm) (Beber et al., 2019; Cannon et al., 2023). Septins’ persistence lengths are approximately one and three orders of magnitude lower than those of actin filaments (∼10–20 μm) and microtubules (∼5,000 μm), respectively (Gittes et al., 1993). Importantly, septin filaments possess both longitudinal and lateral plasticity. The oligomeric interfaces of human septins can compact and expand in a nucleotide-dependent manner (GTP/GDP) (do Vale Castro et al., 2020) and can hinge up to 30° between the terminal subunits of their protomeric oligomers (Sirajuddin et al., 2007). Septin filaments also interact laterally through C-terminal coiled coils of variable length that extend sideways from the linear multimer (Bertin et al., 2008; Szuba et al., 2021). Resembling the side chains of bottlebrush polymers used in elastic biomaterials and electronics (Hu et al., 2025), these C-terminal extensions may enable septin filaments to expand, contract, and rearrange lateral contacts within filament bundles in response to intracellular forces, assembling into mechanically resilient networks (Szuba et al., 2021). In vitro studies show that SEPT9-containing octamers form spirals with a range of curvatures on membrane bilayers, which are not observed with SEPT2/6/7 hexamers, indicating that SEPT9 may enhance the flexibility of septin structures (Reese et al., 2026, *Preprint*). Overall, the plasticity of septin filaments makes them well suited to absorb and dissipate mechanical strain.

Septins may absorb forces not only on their own as deformable semi-elastic polymers, but also as components of actomyosin networks. Actin-binding and crosslinking proteins (e.g., anillin, filamin, fascin) modify the rheological properties and behavior of actin networks in response to mechanical strain (Falzone et al., 2012; Falzone et al., 2013; Gardel et al., 2004; Gardel et al., 2006). Depending on the strength of interaction with actin filaments, protein crosslinkers can dynamically rearrange in response to stress, impacting actin network mechanics. Septins crosslink actin filaments via SEPT9 (Dolat et al., 2014; Iv et al., 2021; Martins et al., 2023; Smith et al., 2015) and therefore may affect the response of actin networks to mechanical stress. Previous work showed that septin complexes crosslink actin filaments in vitro into curved and circular bundles (Mavrakis et al., 2014), indicating that septins can reduce the rigidity of actin polymers, possibly by inducing conformational changes and flexible links that enable bending without breaking. Therefore, septin presence in actomyosin networks may reduce flexural rigidity, and thereby minimize severing under higher mechanical loads. In retrospect, this function of septins may account for the disassembly and disorganization of highly contractile actin networks such as actin stress fibers and cytokinetic rings, which have been extensively reported in a diversity of cell types and organisms (Spiliotis and Nakos, 2021).

During confined cell migration, the nucleus is subjected to contractile, tensile, and hydrostatic forces that enable translocation through spaces smaller than its size (McGregor et al., 2016). Migratory cells adapt different strategies for threading their nucleus through tight spaces, and they have mechanisms for repairing nuclear envelope rupture and DNA damage. However, mechanisms that cushion the nucleus from the mechanical impact of rising intracellular forces remain poorly understood. The intermediate filament protein vimentin, which is upregulated in invasive cancer cells undergoing epithelial-to-mesenchymal transition, forms a perinuclear cage that protects against nuclear deformation (Patteson et al., 2019). The microtubule network also provides a cushion around the nucleus, bearing the load of contractile forces and crosstalking with the actomyosin cytoskeleton by feedback signaling (Ju et al., 2024). Vimentin, which associates with microtubules via plectin, and the microtubule-associated proteins CLASP reinforce the microtubule network to withstand higher intracellular forces (Schmidt and Stehbens, 2024). While vimentin can absorb contractile forces on its own or in association with microtubules, it is also conducive to tensile force transmission, promoting the traction forces of cell migration (Alisafaei et al., 2024; Guo et al., 2025).

Septins emerge as cytoskeletal polymers that protect the nucleus by relieving tensile actomyosin forces in a manner, which is distinct from vimentin. During confined 3D cell migration, we found that septins do not form a perinuclear basket like vimentin but instead co-align with actomyosin cables that surround the nucleus axially in the apicobasal direction of movement, and connect with focal adhesions. On 2D substrates, septin depletion increases actin stress fiber severing at sites proximal to focal adhesions in response to myosin II hyperactivation. Notably, severing occurs preferentially on septin-free actin stress fibers, which retract faster than septin-decorated actin filaments, indicating higher tension. Taken together with an increase in nuclear envelope rupture events in septin-depleted cells, these results suggest that septins buffer the actomyosin forces that move the nucleus forward during confined migration. Interestingly, SEPT9 levels on actin cables increase along with myosin regulatory light chain phosphorylation with increasing confinement (narrower pores) and higher tensile stress (Fig. 2, F and G). This might be a mechanical adaptive response for threading the nucleus forward by a controlled and sustained pulling, where forces are uniformly spread and balanced (Fig. 7)—analogous to how the suspension cables of a hot air balloon distribute lifting forces evenly across the passenger basket. This type of nuclear movement may prevent forces from concentrating focally at sites of the nuclear envelope, which can cause nuclear rupture and DNA damage. Septoactin cables, however, surround the nucleus without making any ostensible end-on contacts, suggesting that septins may buffer force waves that sweep tangentially along the nuclear envelope (i.e., perpendicular to the nuclear radius). These forces may work synergistically with forces generated by motors linking the nuclear envelope to the microtubule network (Ju et al., 2024; Tagay et al., 2023), which along with vimentin is enriched at the leading edge of the migrating nucleus in the Transwell pore. More broadly, SEPT9’s force relieving function may operate in parallel with—and is not mutually exclusive of—its roles in actin stress fiber crosslinking, focal adhesion turnover, and/or ECM degradation (Dolat et al., 2014; Hecht et al., 2024; Marcus et al., 2019; Martins et al., 2023; Okletey et al., 2023; Shankar et al., 2010).

Septins localize preferentially to membrane domains experiencing elevated contractile force and tension, such as the cleavage furrow and cell junctions (Carim et al., 2020; Kim et al., 2023; Naydenov et al., 2025; Shindo and Wallingford, 2014), as well as regions where force distribution and buffering are critical—including T cell mid-zones that suppress protrusive activity (Tooley et al., 2009) and septoactomyosin cages that constrain the motility of pathogenic bacteria (Van Ngo and Mostowy, 2019). The preferential association of septins with domains of micron-scale membrane curvature may serve a similar protective function by relieving tension from membrane stretching at the base and neck of membrane protrusions, or by counteracting turgor pressure at fungal protrusions such as the hyphae of *Aspergillus* and the appressorium of the rice blast fungus (Cannon et al., 2017; Eisermann et al., 2023).

In work published concurrently with this article, Utgaard et al. discovered that septins accumulate on the cell cortex in response to local mechanical compression and signaling (Utgaard et al., 2026). Our combined results suggest that septins can absorb mechanical strain in the membrane, potentially acting as a relief valve for the cytoskeleton and membranes that is conserved across diverse cell types and biological systems. Future work will explore whether tension and specific tension thresholds are critical determinants of septin assembly at membrane domains and actomyosin filaments.

In cancer cells undergoing invasive migration, mechanical tension and stress impact survival not only by compromising nuclear and genomic integrity but also by signaling to apoptotic pathways, which can induce programmed cell death (Kumari et al., 2025; Lim et al., 2016; Mammel and Hatch, 2022; de Freitas Nader et al., 2021). While external microenvironments dictate the types of forces and mechanical stresses induced, how tumor cells mitigate and adapt to these pressures can determine pathogenic course and survival outcomes. Our results suggest that SEPT9_i1 enhances survival by reducing genotoxic mechanical stress. We posit that this function provides a selective advantage for tumor clones and/or subclones suited for invasion and/or growth in specific niches by preserving their highly altered genomes during dissemination through confined tissue spaces. Thus, *SEPT9* amplification may enable metastatic tumor cells to navigate a narrow survival window—buffering actomyosin-generated forces during confined migration to maintain genomic instability within tolerable limits while avoiding lethal genomic catastrophe (Andor et al., 2017; Zhivotovsky and Kroemer, 2004). Interestingly, recent work showed that in nonadherent cancer cells (melanomas), septins scaffold survival signaling pathways on the curvatures of the blebbing plasma membrane (Weems et al., 2023). Taken together with our findings and those by Utgaard et al. (2026), septins appear to adapt prosurvival functions on the actomyosin cytoskeleton and cell membrane in a morphodynamic and mechanoresponsive manner, which in turn may depend on tumor microenvironment and cell–ECM interactions. Overall, SEPT9 amplification emerges as a critical component of the prosurvival toolbox of cancer, raising the value of septins as precision oncology biomarkers and therapeutic targets.

## Materials and methods

### Cell culture

The mammalian cell lines U2OS (ATCC HTB-96) and MDA-MB-231 (ATCC HTB-26) were maintained in DMEM high glucose medium (07777; Sigma-Aldrich). Primary MEFs were derived from *Sept9*^−/−^ mice and control *Sept9*^+/+^ littermates, and immortalized as previously described. They were gifted by Dr. Ernst-Martin Füchtbauer (Aarhus University, Aarhus, Denmark) (Füchtbauer et al., 2011). All media were supplemented with 10% FBS (FR-0500-A; Atlas Biologicals) and 1% penicillin–streptomycin–kanamycin solution (PSK). Cells were maintained in culture at 37°C in a humidified incubator with 5% CO_2_. Cultures were maintained at 70% confluence and subcultured at a 1:5–1:10 ratio every 4 days using 0.05% trypsin–EDTA (25300-054; Gibco).

Prior to experiments, cells were seeded on either glass coverslips (22 × 22 mm, No. 1.5; 48366-227; VWR), which were coated with type I collagen (PureCol type I bovine collagen; 5005; Advanced BioMatrix), or transparent polyester (PET) membrane Transwell inserts (Corning) coated with Matrigel and laminin. Glass coverslips were coated with collagen at a concentration of 2.5 μg/ml for 5 min at room temperature, and subsequently were air-dried for 30 min and exposed to UV light for 30 min in a biosafety cabinet to ensure sterilization. The PET membranes of Transwell inserts were coated on both sides with 200 μl of ice-cold ∼10 mg/ml Matrigel (356237; Corning) and 0.2 mg/ml laminin (L2020; Sigma-Aldrich) and incubated at 4°C with gentle rocking for 30 min to ensure even deposition. Excess Matrigel was aspirated, and membranes were polymerized at 37°C for 30 min in a cell culture incubator prior to cell seeding. Membranes were then hydrated on both sides with DMEM high glucose medium supplemented with 10% FBS and 1% PSK.

For experiments performed on glass coverslips, cells were detached using 0.05% trypsin–EDTA for 5 min at 37°C, neutralized with complete DMEM (DMEM high glucose medium, 10% FBS, and 1% PSK), and collected by centrifugation at 425 rcf for 5 min. The cell pellet was resuspended in fresh complete DMEM, and viable cells were counted using an automated cell counter Countess 3 (Thermo Fisher Scientific) with 0.4% (wt/vol) trypan blue exclusion (25-900-Cl; Corning). Cells were seeded at a density of 2 × 10^5^ cells per coverslip and allowed to adhere and recover for 12–24 h before transfection. For experiments using 24-mm Transwell membrane inserts, cells were seeded at a density of 1 × 10^6^ cells per insert.

For live-cell imaging experiments, cells were seeded onto collagen-coated 35-mm glass No 1.5 bottom dishes (P35G-1.5-20-C; MatTek) and switched to phenol red–free HyClone DMEM/High Modified media (SH30284.01) prior to transferring to the microscope’s stagetop incubator. Live imaging of cells during confined migration through the pores of Transwell membranes was performed by cutting a rectangle strip from the Transwell’s membrane insert and immobilizing it on the glass portion of a MatTek 35-mm glass-bottom dish using a 4-well insert with an adhesive biocompatible bottom surface (80466/80469; ibidi), which was placed on top of the filter and filled with HyClone DMEM/High Modified media.

### Transfection

Cells were transfected transiently with the plasmids pEGFP-C2_SEPT9_i1 (plasmid #71609; Addgene) (Connolly et al., 2011), LAP2 Full I pAcGFP-N1 monomeric GFP (a gift from Dr. Eric Schirmer, The University of Edinburgh, Edinburgh, UK; #62044; Addgene plasmid) (Zuleger et al., 2011), pEGFP-MRLC1 T18D, S19D (a gift from Dr. Tom Egelhoff, Cleveland Clinic, Cleveland, OH, USA; plasmid # 35682; Addgene) (Beach et al., 2011), or pGFP-farnesyl-C3 with A206K mutation in GFP to prevent dimerization (mGFP-farn; a gift from Dr. Betty Eipper, University of Connecticut Health, Farmington, CT, USA) (Francone et al., 2010) using Lipofectamine 2000 transfection reagent (11668019; Invitrogen) according to the manufacturer’s instructions with minor optimization. Transfections were performed on 35-mm dishes using Opti-MEM I Reduced Serum Medium (31985070; Gibco). For plasmid DNA transfection, DNA–lipid complexes were prepared immediately before use. For each dish, 1.5 µg of plasmid DNA was diluted in 120 μl of Opti-MEM I, and 4 μl of Lipofectamine 2000 was diluted in 120 μl of Opti-MEM I in a separate tube. After a 5-min incubation at room temperature, the diluted DNA and Lipofectamine 2000 were combined, gently mixed, and incubated for 20 min at room temperature. Immediately before adding the complexes, the culture medium was replaced with fresh DMEM high glucose medium without antibiotics. The DNA–Lipofectamine 2000 complexes were then added dropwise to the cells and mixed by gently swirling the dishes. Cells were incubated with the complexes at 37°C in a humidified atmosphere containing 5% CO_2_ for 6 h, after which the medium was replaced with complete DMEM.

### Generation of stable CRISPR/Cas9 genome-edited cells

To generate cell lines with stable depletion of SEPT9_i1, a guide RNA (gRNA) targeting the N-terminal segment of SEPT9 isoform 1 was designed within exon 2. The gRNA oligonucleotides with forward primary sequence 5′-CAC​CGC​ACG​CGG​ACC​TCC​AGT​GGC-3′ (Sept9V1-F2) and reverse primer sequence 5′-AAA​CGC​CAC​TGG​AGG​TCC​GCG​TGC-3′ (Sept9V1-R2) were annealed and cloned into the plasmid backbone of the pSpCas9(BB)-2A-Puro (PX459) vector (48139; Addgene), which expresses a Cas9/gRNA cassette, following standard cloning procedures. Correct insertion and sequence integrity were confirmed by sequencing.

U2OS and MDA-MB-231 cells were maintained under standard culture conditions (37°C, 5% CO_2_) in appropriate complete growth medium as described in the Cell culture section. For transfection, cells were seeded 1 day prior to transfection to reach ∼70–80% confluence and were then transfected with the pSpCas9(BB)-SEPT9_i1 gRNA plasmid using Lipofectamine 2000 according to the manufacturer’s instructions, with plasmid DNA and transfection reagent amounts optimized for each cell line. After 4–6 h, the transfection medium was replaced with fresh complete medium, and cells were allowed to recover overnight.

To enrich for transfected cells and facilitate stable line generation, transfected cultures were subjected to selection with puromycin (1 μg/ml) to eliminate nontransfected cells within 48 h. Following 2 days of puromycin selection, surviving cells were trypsinized and reseeded at very low density into 10-cm dishes to allow the formation of individual colonies derived from single cells. Colonies were manually isolated using a sterile filter paper with trypsin and transferred individually into the wells of a 48-well plate, where they were cultured for ∼2 wk with changes of media every 2–3 days. Clonal populations were expanded stepwise into larger dishes. Once sufficient cell numbers were obtained from each clone, whole-cell lysates were prepared and SEPT9_i1 protein levels were assessed by western blot using an isoform-specific anti-SEPT9_i1 antibody, with appropriate loading controls. *SEPT9* exon 2 deletion was confirmed by genomic DNA sequencing using both the sequencing primers 5′-TAG​TAG​GGC​TTG​GGG​GAG​GAT​GTG​TGT​GAA​GG-3′ (SeqCRISPseptV1F) and 5′-ACA​CAC​GGG​CTT​GCT​CCC​TGA​GCC​TGG​GGG​TG-3′ (SeqCRISPseptV1R). The notation CRISPRi was used for cell clones with incomplete deletion due to the presence of mutliple *SEPT9 *gene copies.

### Confined cell migration and myosin hyperactivation assays

In the confined cell migration assays, we used the following Corning Costar Transwell plates: 24-mm Transwell with 3.0-µm pore polycarbonate membrane insert (3452; Corning), 6.5-mm Transwell with 5.0-µm pore polycarbonate membrane insert (3421; Corning), and 24-mm Transwell with 8.0-µm pore polycarbonate membrane insert (3428; Corning). These were coated on both sides with Matrigel and laminin as described above. Two million cells (per 24-mm Transwell with 3.0-µm-wide pores), one million cells (per 24-mm Transwell with 8.0-µm-wide pores), and 0.5 million cells (per 6.5-mm Transwell with 5.0-µm pores) were resuspended in 1.5 ml serum-free medium and seeded directly onto the upper side of the Transwell membrane. The lower chamber received 2 ml of medium supplemented with 50 ng/ml EGF (PHG0311; Gibco) to establish a chemotactic gradient. Cells were allowed to migrate for 12 h (8-µm-wide pores), 24 h (5-µm-wide pores), or 36 h (3-µm-wide pores) at 37°C and 5% CO_2_.

After incubation, cells were washed twice with PBS to remove nonadherent cells and debris. Membranes were fixed in 4% PFA for 15 min at room temperature, quenched with 0.4% ammonium chloride for 10 min to reduce autofluorescence, and permeabilized as needed. Inserts were carefully removed from the Transwell plate, and membranes were mounted onto Parafilm for subsequent immunofluorescence staining.

For myosin II hyperactivation assays, cells were plated on collagen-coated glass coverslips or glass-bottom dishes as described above, and cultured for 24 h prior to treatment with calyculin A (10 nM) or equal volume of DMSO (carrier control). Calyculin (Calyculin A from Discodermia calyx; In Solution, >98%) was purchased from Sigma-Aldrich (5.08226) and reconstituted with DMSO at a stock concentration of 100 μM and kept at −20°C after aliquoting. Cells were incubated with calyculin A for 15 or 30 min prior to fixation and permeabilization for imaging, or imaged live after 5 min of addition to glass-bottom dishes. Inhibition of myosin II activity while treating with calyculin A was performed by incubating cells with media containing 5 μM para-amino-blebbistatin (22699; Cayman Chemical Company) or 10 μM Y27632 (hydrochloride; 10005583; Cayman Chemical Company) for 45 min prior to the addition of calyculin.

To assess the effect of myosin II inhibition on U2OS transmigration, cells were plated for 6 h on Transwell polycarbonate membranes containing either 3-μm- or 8-µm-wide pores. Both sides of each membrane were coated with Matrigel and laminin as previously described. For experiments using 8-µm-wide pores, 0.5 million cells were seeded onto the apical side of the membrane for 6 h, and blebbistatin was subsequently added to both the upper and lower chambers for 12 h in the presence of an EGF gradient (see above). For experiments using 3-µm-wide pores, one million cells were seeded onto the apical side of the membrane for 6 h prior to adding blebbistatin to both upper and lower chambers of the Transwell insert for 24 h in the presence of an EGF gradient (see above). Following transmigration in the presence of blebbistatin, cells were fixed with PFA and stained with DAPI and phalloidin. The percentage of transmigrated cells was calculated as the number of cells on the basal side of the membrane divided by the total number of cells counted on both the apical and basal sides of each field of view.

### Cell viability assay

Cell viability following migration through the pores of the Transwell polycarbonate membranes was evaluated using a trypan blue exclusion assay. Briefly, two Transwell membranes (3-µm pore size) were used per sample replicate. A total of 2 × 10^6^ cells were seeded onto each Transwell membrane and stimulated with EGF (50 ng/ml) to promote directed migration. After 36 h of incubation, cells that had traversed the membranes were recovered from the lower chamber by trypsinization, and collected in culture medium. Cells from the two membranes were pooled and centrifuged at 376 rcf. The resulting cell pellet was gently resuspended in 30 μl of PBS and subsequently diluted 1:1 (vol/vol) with 0.4% trypan blue solution (Invitrogen) for viability assessment. Stained cell suspensions were immediately loaded onto disposable Countess cell counting slides and analyzed using Countess 3 Automated Cell Counter (Thermo Fisher Scientific).

The instrument showed reproducible image-based discrimination of viable (unstained) and nonviable (blue-stained) cells. For each condition, at least three independent biological replicates were quantified, and each replicate measurement represented the mean of three technical readings. The percentage of dead cells was calculated as Cell death (%) = (Number of trypan blue–positive cells/Total number of cells counted) × 100.

### Immunofluorescence

Cells were cultured on the collagen-coated coverslips and subsequently fixed with 4% PFA (Cat #15710; Electron Microscopy Sciences) in PBS for 15 min at 37°C following two PBS washes. To quench residual aldehyde groups, cells were incubated with 0.4% ammonium chloride (Cat #A9434; Sigma-Aldrich) for 5 min at room temperature. Permeabilization was performed using 0.2% Triton X-100 (Cat #X100; Sigma-Aldrich) in PBS for 20 min at room temperature.

After permeabilization, cells were blocked in blocking buffer containing 2% BSA (Cat #A9647; Sigma-Aldrich), and 0.1–0.2% Triton X-100 in PBS for 30 min. The samples were washed twice with PBS, blocked in 2% BSA in PBS for 30 min, and incubated with primary antibodies diluted in blocking buffer overnight at 4°C. After primary antibody incubation, coverslips were washed and then incubated with appropriate fluorescently labeled secondary antibodies for 1 h at room temperature in the dark.

Following immunostaining, coverslips were washed in PBS, stained with DAPI as necessary, and mounted onto frosted microscope slides (Cat #1304W; Globe Scientific) using FluorSave reagent (345789; Millipore). The slides were stored at 4°C and protected from light until imaging.

### Antibodies and fluorescent probes

The following antibodies were used for immunofluorescence microscopy and western blots: rabbit anti-SEPT9 (1:200 10769; Proteintech), rabbit anti-SEPT7 (1:500, 18991; IBL America), rat anti-SEPT9 isoform 1 4D2A5 (1:100, CEC-048; Cell Engineering Corporation), mouse anti-paxillin (1:400, 610051; BD Transduction Laboratories; clone 349), mouse monoclonal anti-phospho-Histone H2A.X (Ser139) clone JBW301 (1:500, 05-636-I; MilliporeSigma), mouse anti-nuclear pore complex clone 414 (1:400, N8786; MilliporeSigma), rabbit anti-zyxin (1:300, 10330; Proteintech), mouse anti-myoIIA (1:100, M8064; MilliporeSigma), rabbit anti-phospho-myosin light chain 2 (Thr18/Ser19) (1:100, 3674; Cell Signaling), rabbit anti-vimentin (1:300, 10366; Proteintech), and mouse anti-GAPDH (1:1,000, Nb600-52SS; Novus).

The following fluorescent antibodies were used for immunodetection of primary antibodies in immunofluorescence experiments: Alexa Fluor 488 AffiniPure donkey anti-rat IgG (H+L) (712-545-153; Jackson ImmunoResearch), Alexa Fluor 488 AffiniPure F(ab)_2_ donkey anti-rabbit IgG (H+L) (712-545-152; Jackson ImmunoResearch), Alexa Fluor 488 AffiniPure F(ab)_2_ donkey anti-mouse IgG (H+L) (715-546-150; Jackson ImmunoResearch), Alexa Fluor 555 AffiniPure F(ab)_2_ donkey anti-rabbit IgG (H+L) (711-565-152; Jackson ImmunoResearch).

Actin was labeled in fixed cells with AF488 phalloidin (23153; AAT Bioquest) and phalloidin-iFluor 647 (ab176759; Abcam), and in live cells with SPY555-actin (CY-SC202; Cytoskeleton), SPY555-FastAct (CY-SC205; Cytoskeleton), or SPY650-FastAct_X (CY-SC502; Cytoskeleton). Cell nuclei were stained with DAPI (1 µg/ml; Thermo Fisher Scientific) in fixed cells and Hoechst 3342 (Invitrogen) in live cells.

For western blots, the following dye-conjugated secondary antibodies were used: IRDye 680RD goat anti-rat (925-68076; LI-COR Biosciences), IRDye 800CW donkey anti-rabbit (926-32213; LI-COR Biosciences), and IRDye 680 donkey anti-mouse (926-32222; LI-COR Biosciences).

### Western blots of cell extracts

MDA-MB-231, U2OS, and their respective SEPT9-deficient cell lines were cultured in 60-mm tissue culture dishes at a density of 1 × 10^6^ cells per plate for 24 h under standard growth conditions. Following incubation, cells were washed once with ice-cold PBS and lysed on ice for 30 min in lysis buffer containing 150 mM NaCl, 50 mM Tris-HCl (pH 8.0), 20 mM NaF, and 1% NP-40, supplemented with cOmplete Mini EDTA-free protease inhibitor cocktail (Cat #11836170001; Roche). Lysates were mixed gently by pipetting to ensure homogeneity and then subjected to two cycles of sonication (10 s on, followed by 5 min off) to disrupt cellular membranes and shear genomic DNA. After sonication, samples were centrifuged at 13,523 rcf for 15 min at 4°C, and the clarified supernatants were collected as total protein extracts. Protein concentration was determined using a Pierce BCA protein assay kit (Bio-Rad), and equal amounts of protein were mixed with 5X Laemmli sample buffer and boiled for 5 min at 95°C to denature the proteins.

Samples were separated by hand-cast SDS–PAGE and subsequently transferred to nitrocellulose membranes using a wet transfer apparatus. Membranes were blocked for 1 h at room temperature in blocking buffer (2% nonfat dry milk in TBS-T: 20 mM Tris-HCl, 150 mM NaCl, 0.1% Tween-20) to prevent nonspecific binding.

The membranes were incubated overnight at 4°C with primary antibodies diluted in blocking buffer, followed by incubation with appropriate infrared dye–conjugated secondary antibodies (IRDye 800CW and IRDye 680RD; LI-COR Biosciences) for 1 h at room temperature. After washing, protein bands were visualized and quantified using Odyssey CLx Infrared Imaging System (LI-COR Biosciences) according to the manufacturer’s instructions.

### Microscopy and image processing

#### Super-resolution spinning-disk confocal microscopy

Imaging of fixed cells in confined migration assays were performed with a NIKON Ti2E motorized microscope with a super-resolution W1-SoRa Yokogawa spinning-disk system equipped with a Hamamatsu ORCA-Fusion BT back-thinned camera, and an environmentally controlled enclosure (Okolab) for adjustment and maintenance of temperature at 37°C, humidity, and CO_2_ during live-cell imaging. Images were acquired with a CFI60 Plan Apochromat Lambda D 60X Oil, N.A. 1.42, DIC, and a SOLA Light Engine Gen III with 350- to 680-nm unfiltered light output and full complement of excitation/emission filter sets, and a laser illumination LUN-F-XL output with 405-nm (50 mW), 488-nm (60 mW), 561-nm (50 mW), and 640-nm (40 mW) laser lines.

Following identification of cell regions of interest using the DAPI fluorescence, super-resolution imaging was conducted by setting Observation Mode to Confocal SoRa with a digital 2.5× magnification and an axial step size of 0.1–0.2 µm (1,152 × 1,152 pixels per slice, 12-bit depth, pinhole 1 AU) for acquisition of a z-series stack, which ensured high-resolution sampling along the z axis and accurate 3D reconstruction of the cellular structures within the pores. Laser power was set to 10% to minimize photobleaching, with dwell time of 1.6 µs per pixel. Images were acquired using Nikon NIS-Elements C software (v. 6.4, Nikon Instruments).

Raw z-stacks were processed in Nikon NIS-Elements Advanced Research software (v. 6.4). A Gaussian noise reduction filter (σ = 1.0) was applied first to minimize background signal and enhance signal-to-noise ratio, followed by blind deconvolution (10 iterations, Richardson–Lucy algorithm) to enhance resolution and contrast. Processed stacks underwent 3D volume rendering (isosurface projection, threshold 20–50% max intensity) for pore-cell reconstruction, enabling detailed visualization and structural assessment of cells occupying or migrating through the membrane pores.

#### Wide-field deconvolution microscopy

Imaging of fixed cells on 2D collagen-coated coverslips was performed with a NIKON Ti2E motorized wide-field/deconvolution microscope system equipped with a Hamamatsu ORCA-Fusion BT back-thinned camera, Plan Apochromat Lambda D 60× oil immersion objective lens (N.A. 1.42) with phase-contrast and DIC condensers and prisms, and a SPECTRA III illumination source for excitation at 390-, 440-, 475-, 510-, 555-, 575-, 637-, and 748-nm wavelength band-pass filters. Images were acquired with Nikon Elements software with postprocessing (denoising; LIM 2D/3D deconvolution) and analysis tool on a HP Z4 advanced imaging workstation. Samples were illuminated under conditions optimized to prevent photobleaching while ensuring adequate signal-to-noise ratio. Image stacks were acquired with 0.1- to 0.2-µm z-step intervals to capture the full 3D structure of the specimen. Images were denoised and deconvolved, as indicated, using Nikon Elements software.

### Image analysis and quantifications

#### Quantification of actin cable decoration with zyxin, pMLC2, and SEPT9, and contact with paxillin

Images were first subjected to a noise reduction procedure and subsequent deconvolution using NIS-Elements software (Nikon) to improve signal-to-noise ratio and spatial resolution prior to quantitative analysis. Quantification of zyxin with actin filaments was conducted following 3D image reconstruction of cells using NIS-Elements software (Nikon Instruments). 3D image reconstructions were then generated from SoRa z-stacks comprising 20–30 optical sections using the “Show 3D View” function, thereby enabling accurate visualization and measurement of filament geometry in three dimensions. Zyxin fluorescence signals colocalizing with actin cables were identified, excluding signals located at filament termini, and individual actin cables with zyxin fluorescence were scored as zyxin-positive. Actin cables with end-on contacts and overlaps with paxillin were also recorded in 3D volumes of cell regions confined in pores. The number of zyxin-, pMLC2-, or SEPT9-positive cables, and paxillin-capped actin cables per cell was recorded, and calculated as the percentage of the total number of actin cables per cell after recording the absolute number of the latter. Percentiles for each cell were imported into GraphPad Prism (GraphPad Software) for statistical analysis and graphing.

Quantification of actin cable coverage by pMLC2 or SEPT9 as the mean percentage of actin filament length with pMLC2 or SEPT9 per cell was done in the Claude CODE AI app with Python 3 after loading the raw 3D image stacks (nd2 files). Actin cables were segmented by applying a 3D Sato tubeness filter (σ = 1.0–2.0) to the actin channel, thresholding at the 90th percentile to create a binary mask, and then skeletonizing it to single-voxel-wide centerlines. The skan library extracted actin segments and measured their lengths in micrometers. For pMLC2 and SEPT9, binary masks were created the same way (90th-percentile threshold) and dilated by one voxel to correct for shifts/registration errors. For each skeleton, the percentage of voxels overlapping each marker mask was calculated using a fast vectorized counting method (numpy.bincount). From this, the percentage of filaments (>2 µm) with marker coverage was derived as mean value per cell.

### Quantification of actin filament number, length, severing, bending, and retraction rates

Quantification of actin filament number and length was done using the Nikon Elements tool “3D Measurement” from the “Measure” software function, which enabled the marking of each actin cable, and the lengths were measured using the “3D Length” function. Results per each confined region were compiled in Microsoft Excel, and imported into GraphPad Prism 6 for data analysis, graph generation, and statistical analysis.

Quantification of actin bending was performed in the Claude Code desktop app with the Python 3 image processing and analysis tools: nd2, scikit-image, skan, NumPy, pandas, and SciPy. For each cell, the F-actin volume was extracted from the nd2 file and smoothed with a 3D Gaussian filter (scaled for voxel anisotropy). The image was segmented using Li’s thresholding (Otsu’s method), and small objects (<100 voxels) were removed. The resulting mask was skeletonized into single-voxel-wide filaments using a standard 3D thinning algorithm, and skan was used to build the skeleton graph in physical units (µm), excluding closed-loop branches. For each filament with length ≥2 µm, arc length (total path length) and end-to-end distance (straight-line distance between endpoints) were measured, and the LI was derived as LI = end-to-end distance/arc length, where LI = 1 means perfectly straight, and lower values indicate more coiling. Filaments with LI <0.5 were empirically classified as highly “curved.”

In images of MEF cells cultured in 2D, actin stress fibers were segmented using the auto thresholding function in Fiji/Image J. Broken actin filaments were identified as individual phalloidin-labeled actin stress fibers with at least one discontinuity or gap along their lengths. Buckled actin filaments were identified as phalloidin-labeled actin stress fibers with at least one bent or wavy segment. The number of broken actin and buckled filaments per cell was scored after blinding for experimental conditions. The percentage of broken and buckled actin filaments was calculated as (number of broken or buckled filaments per cell/total filaments per cell) × 100%. Data were compared with control values, and statistical analyses were performed using GraphPad Prism.

In time-lapse images of MEF cells, quantification of actin stress fiber severing sites as septin-coated or septin-free sites was done by applying a minimum of 10 μm length of actin coverage or noncoverage by GFP-SEPT9_i1 signal with respect to the site of breakage, which was visualized by the SPY555-actin fluorescence signal. Sites of severing that localized within a ≥10-μm-long actin segment coated with GFP-SEPT9_i1 were scored as septin-positive, and sites of severing that localized within a ≥10-μm-long actin segment free of GFP-SEPT9_i1 were scored as negative. Following a severing event, retraction rates were measured by generating a thin-sliced ROI along the axis of breakage. The average velocity was calculated from time–distance montages (kymographs) from the initial breakage point to stoppage point or until the stress fiber retracted past the field of view or became ambiguous.

#### Quantification of nuclear areas and nuclear envelope rupture

Nuclear areas were quantified in the Claude Code AI desktop app using Python 3. Nuclear segmentation was performed using the scikit-image library after importing the DAPI channel from a single optical section, which was the second to the most ventral. Images were contrast-stretched by clipping intensities to the 2nd–98th percentile range and normalizing to [0, 1]. Gaussian smoothing was applied with σ = 2.0 pixels (0.22 µm) to reduce noise prior to thresholding. Binary masks were generated using the triangle thresholding method, with Otsu’s method applied as a fallback. Small holes (<500 pixels) were removed, and binary holes were filled using morphological operations, followed by removal of objects below 20 µm^2^. Nuclei were separated using a distance-transform watershed algorithm: the Euclidean distance transform of the binary mask was computed, local maxima were identified as watershed seeds, and the resulting labels delineated individual nuclear boundaries. Nuclei touching the image border were excluded. Nuclear area quantification: Nuclear cross-sectional area (µm^2^) was calculated by multiplying each nucleus’s pixel count by the pixel area (0.01170 µm^2^ pixel^−1^). Nuclei were retained if their area fell within the range of 50–5,000 µm^2^ to exclude debris and cell clusters.

To quantify nuclear rupture events, fluorescence microscopy images of cells stained with DAPI and the mouse mAb 414 against nucleoporins, which outlined the nuclear envelope, were analyzed using Fiji (ImageJ). Images were first imported as multichannel stacks, and each channel was separated to independently evaluate the nuclear membrane continuity that surrounds DNA. Background subtraction and intensity normalization were applied to improve signal-to-noise ratio. Using the nucleoporin channel, the nuclear boundary was visualized and segmented using manual thresholding. Rupture was defined as a discontinuity or loss of membrane signal continuity surrounding the nuclear DAPI mass. The DAPI channel was used to confirm the presence of DNA protrusions extending beyond these discontinuities. For each image, nuclei were counted and categorized as ruptured or intact. Quantification was expressed as the percentage of ruptured nuclei relative to the total number of nuclei analyzed per field of view. Multiple fields were evaluated for each condition, ensuring statistical robustness. Data were compiled and analyzed using Fiji’s measurement tools and exported for statistical processing in GraphPad Prism.

#### Quantification of DNA damage, micronuclei, and nuclear blebs

DNA damage image analysis was performed using FIJI/ImageJ. Individual nuclei were segmented based on DAPI staining, and cells exhibiting at least one large γH2AX focus with surface area larger than 0.4 μm^2^ were classified as γH2AX-positive. The fraction of γH2AX-positive cells was calculated as percentage of γH2AX-positive cells = (Number of γH2AX-positive nuclei/Total number of nuclei analyzed) × 100.

Micronuclei were identified and quantified as DAPI-positive structures separated from the main nuclear mass, with a diameter less than one third of the primary nucleus, and surrounded by a continuous NUP414-positive envelope. The percentage of cells containing micronuclei was calculated as Micronuclei (%) = (Number of cells with micronuclei/Total number of cells) × 100.

Nuclear blebbing analysis was done in cells stained with mAb 414 against nucleoporins and counterstained with DAPI. A cell was scored as positive for nuclear blebbing if the DAPI signal displayed an irregular contour with at least one protruding bleb, which emerged from and/or outlined by a discontinuous or broken nuclear rim as evident by the nucleoporin fluorescence signal. The percentage of cells exhibiting nuclear blebs was calculated per each region imaged (field of view) as Nuclear bleb (%) = (Number of cells with nuclear blebs/Total number of cells) × 100. At least 50 cells from independent biological replicates were quantified per condition.

### Analysis of clinical genomic data

#### Data sources and acquisition

TCGA molecular and clinical data were retrieved from cBioPortal using the R package cBioPortalData. TCGA studies were screened for the availability of discrete GISTIC copy-number profiles, TMB annotations, and corresponding clinical and survival data. For each eligible study, study-level clinical data, *SEPT9* discrete copy-number calls, and TMB values were downloaded via the cBioPortal API and stored locally for downstream harmonization.

#### Data harmonization and quality control

Study-level tables were harmonized into a unified clinicogenomic analysis dataset using a study-aware merge strategy. Copy-number alterations and TMB values were required to be unique per study and sample, and clinical annotations were used to map samples to patients. Overall survival in months was used as the primary endpoint, with one survival record retained per patient.

To prevent duplication across TCGA study variants, sample identifiers appearing in multiple studies were resolved by retaining a single canonical record per sample. Samples with unresolved conflicts or missing key annotations were excluded. The final analysis dataset contained one row per unique tumor sample with consistent copy-number, mutational, clinical, and survival annotations.

#### Tumor-type enrichment of *SEPT9* copy-number alterations

Tumor-type enrichment of *SEPT9* copy-number alterations was evaluated using two binary exposure definitions: (1) gain or amplification (GISTIC ≥ +1) and (2) amplification-only (GISTIC = +2). For each tumor type with sufficient sample size (*N* ≥ 20), enrichment was assessed using Fisher’s exact test by comparing the frequency of the CNA state within the tumor type to all other tumor types combined. P values were adjusted for multiple testing using the Benjamini–Hochberg procedure. Effect sizes were summarized as odds ratios and log2-transformed odds ratios for visualization.

#### Copy number–expression association

To assess whether *SEPT9* copy-number alterations are associated with increased transcript abundance, matched copy-number and mRNA expression data were analyzed from TCGA breast cancer cohort accessed via cBioPortal (Firehose Legacy). Tumors were stratified by SEPT9 GISTIC copy-number state (−2, −1, 0, +1, +2), and *SEPT9* mRNA expression values were extracted as provided by the portal.

Differences in expression across copy-number strata were evaluated using a Kruskal–Wallis ANOVA test. Where indicated, monotonic association between copy-number state and mRNA expression was additionally assessed using the Spearman rank correlation, treating copy-number state as an ordinal variable.

#### Association between *SEPT9* copy number and chromosomal instability

Chromosomal instability was quantified using FGA, defined as the proportion of the genome affected by copy-number gains or losses. Analyses were restricted to tumors with nonmissing FGA values and available *SEPT9* GISTIC copy-number calls.

Tumors were stratified by *SEPT9* copy-number status as gain or amplification (GISTIC ≥ +1) versus copy-neutral tumors. Differences in the distribution of FGA between groups were evaluated using nonparametric density comparisons. As a sensitivity analysis, FGA was reconstructed for the same TCGA-BRCA tumors, and FGA was recalculated after excluding chromosome 17 from both the altered-base numerator and the covered-base denominator. The resulting FGA distributions were compared using a two-sided Wilcoxon rank-sum test. Differences in the distribution of FGA between groups were evaluated using nonparametric density comparisons.

#### Association of *SEPT9* copy number with tumor stage and grade

Tumor stage and histologic grade were obtained from harmonized TCGA clinical annotations and reflect American Joint Committee on Cancer (AJCC) staging criteria. The AJCC stage integrates the extent of the primary tumor, regional lymph node involvement, and distant metastasis to classify disease severity into stages I–IV. For pan-cancer analyses, detailed AJCC substages were collapsed into a simplified categorical variable to enable consistent comparisons across tumor types. Tumor-grade information was derived from pathology-reported histologic-grade annotations. Where available, grades were classified using conventional four-tier systems (G1–G4), reflecting increasing degrees of tumor dedifferentiation and proliferative aggressiveness, with G1 representing well-differentiated tumors and G4 representing poorly differentiated or anaplastic tumors. To ensure interpretability and consistency across cancer types, analyses of tumor grade were restricted to samples annotated as G1–G4, excluding ambiguous or nonstandard categories (e.g., GX or unknown). *SEPT9* copy-number status was defined from discrete copy-number calls and categorized as copy-neutral, gain, or amplification. Associations between *SEPT9* copy-number state and clinical variables were evaluated using contingency table analyses. Differences in the distribution of tumor stage and tumor grade across *SEPT9* copy-number states were assessed using Pearson’s chi-square tests of independence. All analyses were performed in a pan-cancer cohort, and reported P values are two-sided.

#### TMB and hypermutation analyses

TMB was analyzed in the harmonized TCGA clinicogenomic dataset (see Source Data TumorMutationBurdenAnalysis). To avoid mixing incompatible TMB annotations, analyses were restricted to samples with nonmissing numeric TMB values. *SEPT9* copy-number status was defined from GISTIC discrete calls, comparing tumors with gain or amplification (cna_discrete ≥ +1) to copy-neutral tumors (cna_discrete = 0); deletion states were excluded from these comparisons. To define hypermutation in a tumor type–adjusted manner, high TMB was defined within each cancer type as tumors in the top 20% of the TMB distribution (80th percentile cutoff), computed for tumor types with ≥20 evaluable samples. Enrichment of high-TMB tumors among *SEPT9*-altered versus copy-neutral tumors was evaluated using Fisher’s exact tests in pan-cancer analyses and within the subset of tumor types previously identified as enriched for *SEPT9* copy-number alteration. To account for tumor-type stratification, Cochran–Mantel–Haenszel tests were additionally performed with cancer type as the stratification variable.

For continuous TMB analyses, TMB was log-transformed as log10(TMB + 0.1). Associations between *SEPT9* status and continuous TMB were tested using linear models including tumor type as a fixed effect (log10(TMB + 0.1) ∼ *SEPT9* status + cancer type). As a tumor-normalized sensitivity analysis, within-tumor–type z-scores of log10(TMB+0.1) were computed and modeled as a function of *SEPT9* status.

### Statistical analyses

Statistical analysis of data and graph generation was performed with GraphPad Prism statistical analysis software. Violin plots display the individual values of all datasets, and indicate the median (black line), and first and third quartiles (colored lines). Dataset groups were first checked for standard deviation (SD) variance using the Prism’s Descriptive Statistics tool and then assessed for normal distribution of variance using the D’Agostino and Pearson and Shapiro–Wilk normality tests. For pairwise comparisons of data with normal distributions, Student’s *t* test was used if SDs were equal, or Welch’s *t* test if SDs were unequal. The Mann–Whitney test was used for non-normally distributed data. For statistical analysis of multiple groups with normally distributed data, a Brown–Forsythe test and a Welch ANOVA test were performed with a post hoc Dunnett T3 test for multiple pairwise comparisons. For data that were not normally distributed, a nonparametric Kruskal–Wallis ANOVA test was performed with post hoc Dunn’s test for multiple comparisons. Statistical analysis across groups with categorical variables was performed with the chi-square test of independence with Yates’ continuity correction, and Fisher’s exact test, which was further adjusted with Bonferroni’s correction for multiple comparisons. Additional statistical tests in the analysis of clinical genomic data were as indicated above. Data with P values lower than 0.05 were considered as statistically significant.

### Online supplemental material

Fig. S1 shows that perinuclear septin cables consist of SEPT9_i1 and SEPT7, and are spatially distinct from the nuclear vimentin basket. Fig. S2 shows septin expression in genome-edited U2OS cells, and zyxin increase in confined SEPT9_i1-depleted U2OS cells. Fig. S3 shows the effect of myosin II inhibition by blebbistatin on the transmigration of U2OS cells through 3-μm- and 5-μm-wide pores. Fig. S4 shows SEPT9 expression and localization in *Sept9*^+/+^ and *Sept9*^−/−^ MEFs, and nuclear aberrations in the absence of confined migration. Fig. S5 shows that unrestricted myosin II contractility impairs nuclear integrity in *Sept9*^−/−^ MEFs. Fig. S6 shows SEPT9 expression and localization in MDA-MB-231 cells during confined migration, and effects of SEPT9_i1 depletion on genomic integrity and cell viability. Video 1 shows perinuclear actin cables surrounding the pore-occupying nucleus and contacting paxillin during 3D confined migration. Video 2 shows perinuclear actin cables at the pore rim closely apposed to the plasma membrane. Video 3 shows that SEPT9_i1 aligns vertically with the actin cables that surround the pore-occupying nuclear mass. Video 4 shows SEPT9_i1-coated actin filament segments retracting more slowly than septin-free segments after severing induced by myosin II hypercontractility. Video 5 shows septin-associated actomyosin cables surrounding the nucleus in a pore-occupying region of an MDA-MB-231 cell. Table S1 shows the source data for the mutation burden analysis, the results of which are cited in Results. Data S1 shows the source data for graphs from Fig. 2. Data S2 shows the source data for the graphs from Fig. S3. Data S3 shows the source data for the graphs from Fig. 3. Data S4 shows the source data for graphs from Fig. 4. Data S5 shows the source data for the graphs from Fig. 5. Data S6 shows the source data for the graphs from Fig. S4. Data S7 shows the source data for the graphs from Fig. S6. Data S8 shows the source data for the graphs from Fig. 6.

## Declaration for use of generative AI-assisted technologies in manuscript preparation

The authors used https://claude.ai/ to edit and refine sentences and grammar, and Claude Code to script and implement Python program–based data analyses.

## Supplementary Material

Table S1 shows the source data for the mutation burden analysis, the results of which are cited in Results.

Data S1 shows the source data for graphs from Fig. 2.

Data S2 shows the source data for the graphs from Fig. S3.

Data S3 shows the source data for the graphs from Fig. 3.

Data S4 shows the source data for graphs from Fig. 4.

Data S5 shows the source data for the graphs from Fig. 5.

Data S6 shows the source data for the graphs from Fig. S4.

Data S7 shows the source data for the graphs from Fig. S6.

Data S8 shows the source data for the graphs from Fig. 6.

SourceData FS2 is the source file for Fig. S2.

SourceData FS6 is the source file for Fig. S6.

## Data Availability

Data are available in the article itself and its supplementary materials.
