# Supplementary material for: Septins buffer actomyosin forces to protect the nucleus from genotoxic mechanical stress
Source: J Cell Biol. 2026 Oct 22;225(12):e202602074. doi: 10.1083/jcb.202602074 (PMC13689686; doi:10.1083/jcb.202602074)
Supplement: Supplementary file 2 [file jcb_202602074_sourcedatafs6.pdf]

Full size gel scans
(Figure S6G)

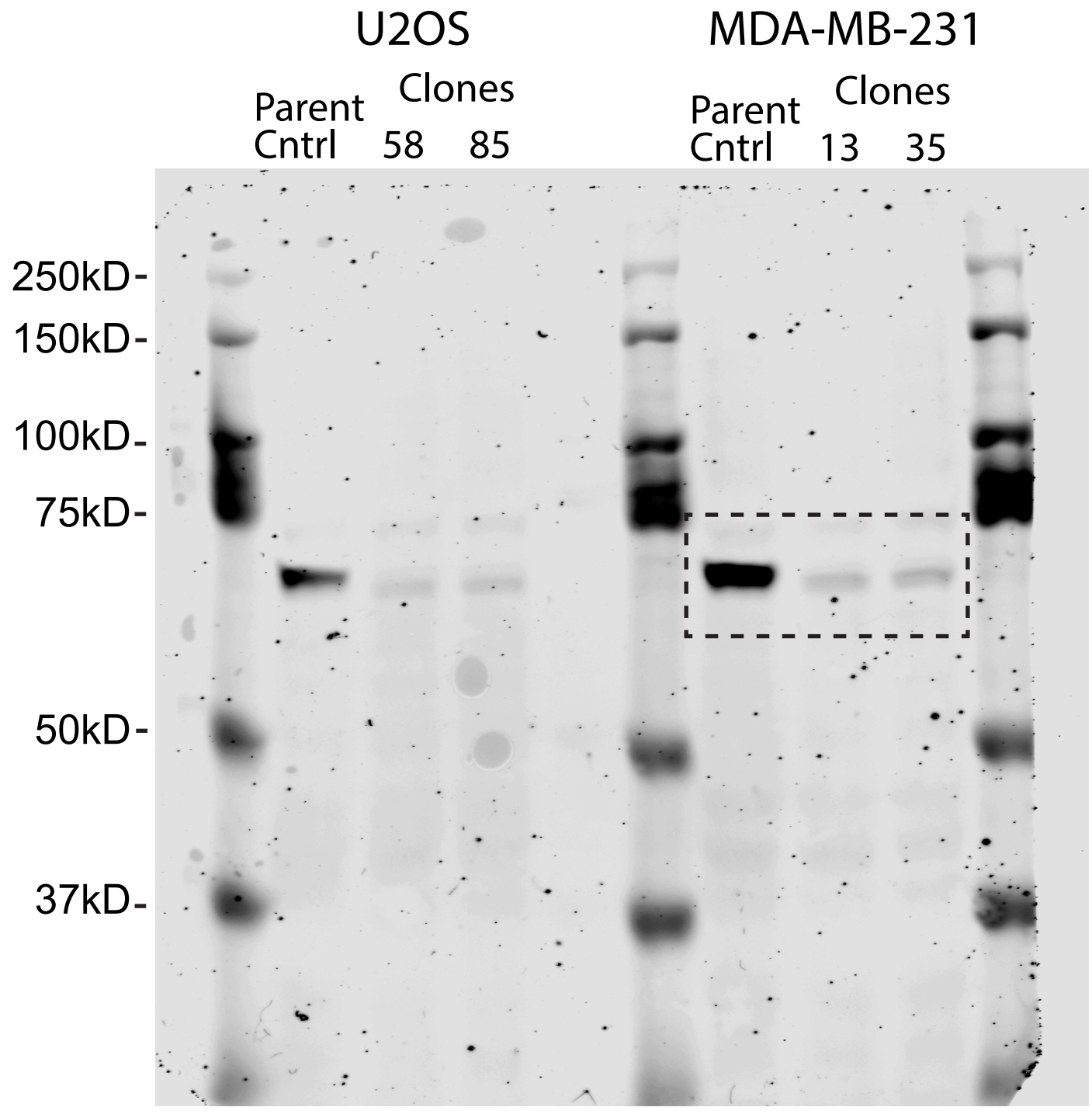

rat anti-SEPT9_i1
western blot

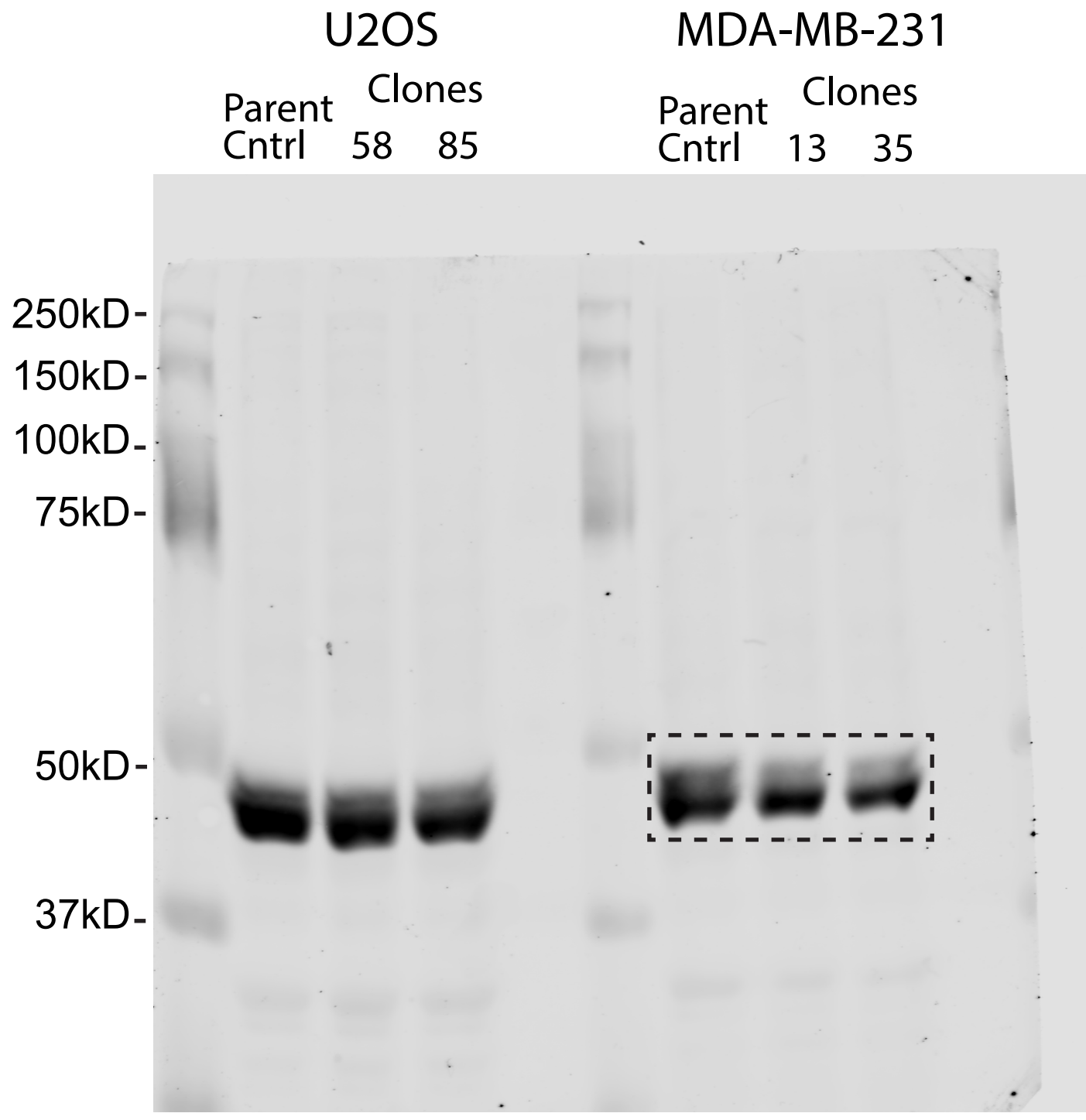

rabbit anti-SEPT7
western blot

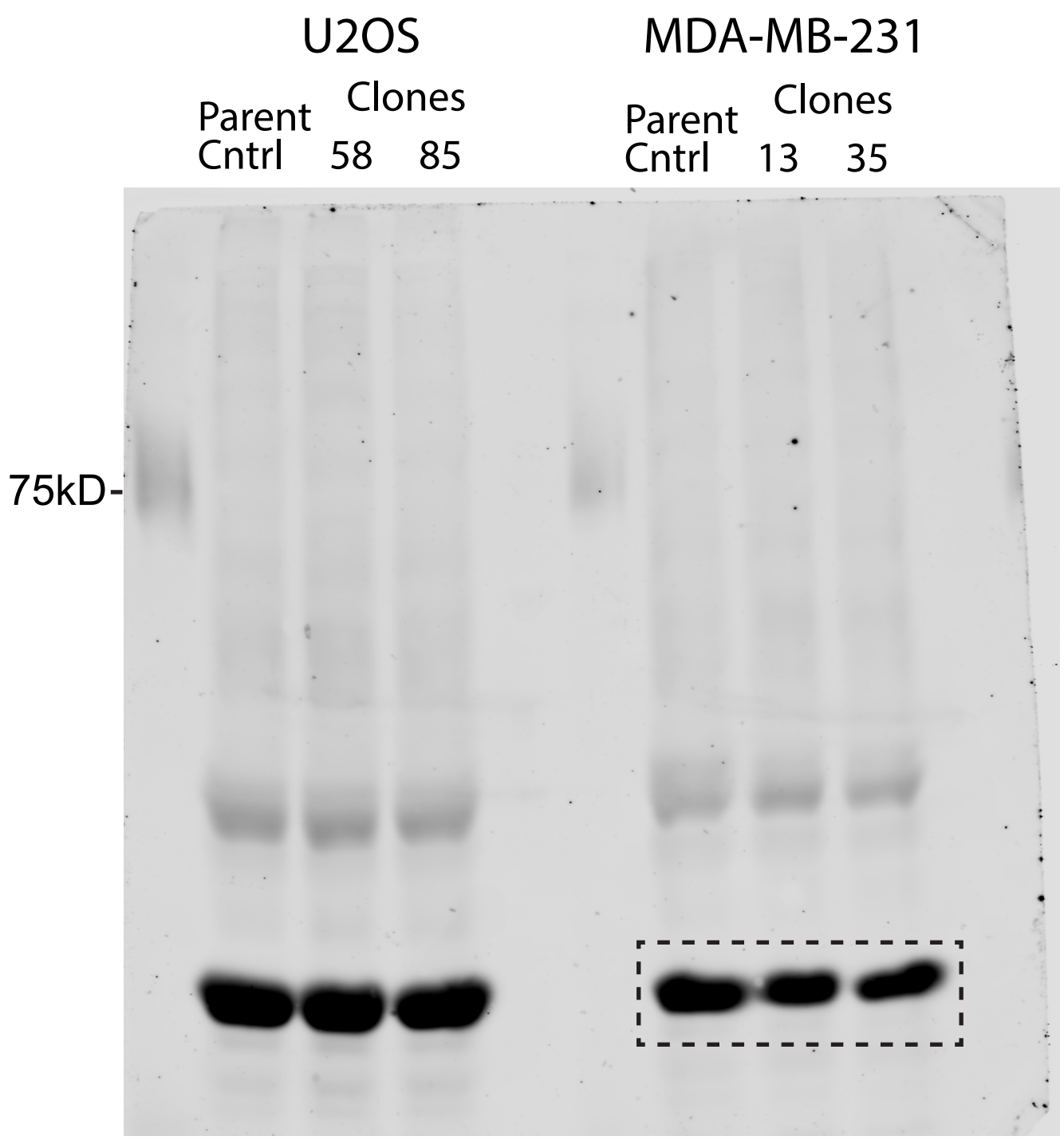

mouse anti-GAPDH
western blot
